# Anti-Candidal Activity of the Parasitic Plant *Orobanche crenata* Forssk

**DOI:** 10.3390/antibiotics10111373

**Published:** 2021-11-09

**Authors:** Floriana D’Angeli, Fiorella Guadagni, Carlo Genovese, Daria Nicolosi, Angela Trovato Salinaro, Mariarita Spampinato, Giuliana Mannino, Debora Lo Furno, Giulio Petronio Petronio, Simone Ronsisvalle, Federica Sipala, Luca Falzone, Vittorio Calabrese

**Affiliations:** 1 Department of Human Sciences and Quality of Life Promotion, San Raffaele Roma Open University, Via di Val Cannuta 247, 00166 Rome, Italy; floriana.dangeli@uniroma5.it (F.D.); fiorella.guadagni@sanraffaele.it (F.G.); 2 InterInstitutional Multidisciplinary Biobank (BioBIM), IRCCS San Raffaele Pisana, Via di Val Cannuta 247, 00166 Rome, Italy; 3 Faculty of Medicine and Surgery, “Kore” University of Enna, Contrada Santa Panasia, 94100 Enna, Italy; 4 Nacture S.r.l, Spin-Off University of Catania, Via Santa Sofia 97, 95123 Catania, Italy; dnicolosi@unict.it; 5 Department of Drug and Health Sciences, University of Catania, Viale Andrea Doria 6, 95125 Catania, Italy; 6 Department of Biomedical and Biotechnological Sciences, Section of Biochemistry, University of Catania, Via Santa Sofia 97, 95123 Catania, Italy; trovato@unict.it (A.T.S.); mariaritaspampinato93@gmail.com (M.S.); calabres@unict.it (V.C.); 7 Department of Biomedical and Biotechnological Sciences, Section of Physiology, University of Catania, Via Santa Sofia 97, 95123 Catania, Italy; giuliana.mannino@unict.it (G.M.); lofurno@unict.it (D.L.F.); 8 Department of Medicine and Health Sciences “Vincenzo Tiberio”, University of Molise, Via Francesco de Sanctis 1, 86100 Campobasso, Italy; giulio.petroniopetronio@unimol.it; 9 Department of Drug and Health Sciences, Section of Medicinal Chemistry, University of Catania, 95125 Catania, Italy; s.ronsisvalle@unict.it (S.R.); sipalafederica@gmail.com (F.S.); 10 Laboratory of Experimental Oncology, Department of Biomedical and Biotechnological Sciences, University of Catania, Via Santa Sofia 97, 95123 Catania, Italy; l.falzone@istitutotumori.na.it

**Keywords:** *Orobanche crenata*, parasitic plant, *Candida* spp., phenotypic switching, biofilm, adhesion, ARPE-19 cells, wound healing, phenols

## Abstract

*Candida albicans* (*C. albicans*) and *Candida glabrata* (*C. glabrata*) are part of the human microbiome. However, they possess numerous virulence factors, which confer them the ability to cause both local and systemic infections. Candidiasis can involve multiple organs, including the eye. In the present study, we investigated the anti-candidal activity and the re-epithelizing effect of *Orobanche crenata* leaf extract (OCLE). By the microdilution method, we demonstrated an inhibitory effect of OCLE on both *C. albicans* and *C. glabrata* growth. By crystal violet and 3-(4,5-dimethylthiazol-2-yl)-2,5-diphenyltetrazolium bromide (MTT) assay, we showed the ability of OCLE to inhibit the biofilm formation and the viability of yeast cells, respectively. By germ tube and adhesion assays, we proved the capacity of OCLE to affect the morphological transition of *C. albicans* and the adhesion of both pathogens to human retinal pigment epithelial cells (ARPE-19), respectively. Besides, by MTT and wound healing assay, we evaluated the cytotoxic and re-epithelizing effects of OCLE on ARPE-19. Finally, the Folin–Ciocalteu and the ultra-performance liquid chromatography-tandem mass spectrometry revealed a high content of phenols and the presence of several bioactive molecules in the extract. Our results highlighted new properties of *O. crenata*, useful in the control of *Candida* infections.

## 1. Introduction

*Candida* is a heterogeneous yeast genus, belonging to the kingdom Fungi. According to the Integrated Taxonomic Information System (ITIS), the genus comprises approximately 59 species (Taxonomic Serial No.: 194591) [1]. *Candida* spp. are generally harmless for the host, being members of the common microbial flora inhabiting the human gut, vagina, and oral cavity of healthy individuals [2]. However, this mutualistic relationship is based on a delicate equilibrium between host and commensals. Accordingly, alterations in the microbiota balance, ecological environment, or host immune defenses promote the switch of *Candida* from non-virulent commensal into an opportunistic pathogen, able to cause infections of varying severity [3].

Among *Candida* species, *Candida albicans* (*C. albicans*) is recognized as the etiological agent involved in the majority of fungal infections in humans [4]. The prominent role of this microorganism in both the community and hospital field is due to its high pathogenic potential. Indeed, *C. albicans* possesses a large variety of virulence factors, including the ability to adhere to host cells and medical devices [5], change its morphology (the so-called yeast-to-hypha transition) [6], produce hydrolytic enzymes [7], and form biofilm [8]. All these features make *C. albicans* a relevant threat to human health, especially for immunocompromised individuals [9]. In the latter, *C. albicans*-related infections can easily degenerate, causing complications sometimes severe enough to be fatal. This is because the immune system of such patients fails to control the proliferation and invasion of opportunistic organisms, leading to an increased risk for invasive candidiasis [10]. In this case, the microorganism leaves the niches in which it is normally located to invade the bloodstream, thus provoking disseminated candidemia [11]. Bloodstream infections constitute a rather severe condition since the blood is an efficient vehicle through which the pathogen can easily reach and infect multiple organs, including the kidney, liver and spleen, myocardium, brain, and eye [12].

In this regard, hematogenous *Candida* dissemination can frequently extend to the ocular district, affecting structures essential for the vision, like the retina and uvea. Therefore, it is not surprising that, in the case of candidemia, *C. albicans* can be responsible for severe ocular infections, including chorioretinitis and endophthalmitis, which, in the absence of prompt and effective treatment, can result in a visual loss in the affected patients [13]. Indeed, in these cases, the intravitreal injection of amphotericin B is the only effective treatment [14]. Interestingly, although immunocompromised individuals are more susceptible to opportunistic infections, rarely, these complications can also arise in immunocompetent patients [15,16,17], exacerbating the risk for ocular candidiasis.

Besides *C. albicans*, non-*albicans Candida* (NAC) species are having an increasing impact on human health [18]. Specifically, *Candida glabrata* (*C. glabrata*) represents one of the most common NAC species isolated in both mucocutaneous and invasive infections [19]. It is worth noting that *C. glabrata* is characterized by an innate resistance to the azoles, which comprise the imidazoles (e.g., clotrimazole, econazole, and miconazole) and triazoles (e.g., fluconazole, voriconazole, and posaconazole) [19,20]. Besides, this microorganism is not able to form hyphae, which instead constitute an important morphologic strategy for *C. albicans* invasion [21]. However, *C. glablata* is also able to invade the bloodstream, especially in case of impairment of anatomical barriers, a condition frequently associated with nosocomial practices [22]. Similar to *C. albicans*, diffuse *C. glabrata* infections can also involve the eye, provoking severe ocular diseases. A case report study highlighted the isolation of *C. glabrata* in a patient that underwent surgical corneal intervention. The patient showed all the predisposing factors to the onset of endogenous endophthalmitis, including old age, immune disorders, and diabetes [23].

This scenario is further complicated by the increasing pharmacological resistance of *Candida* spp. to the most used antifungal agents, such as azoles and echinocandins [24]. Moreover, these antifungal drugs often fail to eradicate the biofilm formed by *Candida* spp., allowing recurrent infections [25]. For these reasons, in the last decade, notable scientific efforts have been dedicated to the discovery and development of new antimicrobial agents, which allow the current therapeutic limits to be overcome. Concerning this, the natural extracts have attracted great attention, since they contain a pool of biologically active molecules [26]. Literature data reported that phytoextracts are endowed with different pharmacological actions, including anti-inflammatory [27], antitumoral [28,29], antioxidant [30,31], and antimicrobial activities [32,33,34]. In this regard, in our previous paper, we highlighted an interesting antibacterial activity of *Orobanche crenata* leaf extract (OCLE) against several clinically relevant bacterial strains. Besides, we also demonstrated a fungicidal effect of the extract on most of the *Candida* spp. tested [35].

*O. crenata* is an edible parasitic plant, known in folk medicine for its numerous beneficial effects on human health [36]. Therefore, based on the promising results previously obtained [35], we proposed to deepen the antifungal activity of this extract, investigating its ability to counteract the growth and biofilm formation of two *Candida* species: *C. albicans* and *C. glabrata*. Furthermore, we also analyzed the capacity of the extract to inhibit the phenotypic switching of *C. albicans* from yeast to hypha and the potential anti-adhesive activity of the two pathogens on the human retinal pigment epithelial cell line (ARPE-19). In addition, given the deleterious effects of *Candida* infection at the ocular level, by wound healing assay, we also tested the possible re-epithelizing action of OCLE on ARPE-19 cells. Finally, we also determined the total phenols content and the phytochemical profile by the Folin–Ciocolteau and ultra-performance liquid chromatography-tandem mass spectrometry (UPLC-Ms/Ms) techniques, respectively.

## 2. Results

### 2.1. Antifungal Activity

Table 1 shows the results of the antifungal effect of OCLE, compared to the reference drug fluconazole. In the assay, 10 different concentrations of the extract, ranging from 0.57 to 293.55 µg/mL, were tested. The minimum inhibitory concentration 50 (MIC_50_) was defined as the lowest extract or drug concentration at which a 50% decrease in turbidity was observed compared with the positive growth control (drug-free medium). The minimum fungicidal concentration 50 (MFC_50_) was defined as the lowest extract or drug concentration at which 50% of the inoculums were killed.

The MIC_50_ values for *C. albicans* and *C. glabrata* were found to be 146.77 and 73.38 µg/mL, respectively. The *Candida* strains were dose-dependent susceptible to the antifungal drug fluconazole, with an MIC_50_ value of 16 µg/mL.

### 2.2. Biofilm Inhibition and 3-(4,5-Dimethylthiazol-2-yl)-2,5-diphenyltetrazolium Bromide (MTT) Reduction Assays

**Figure 1:**
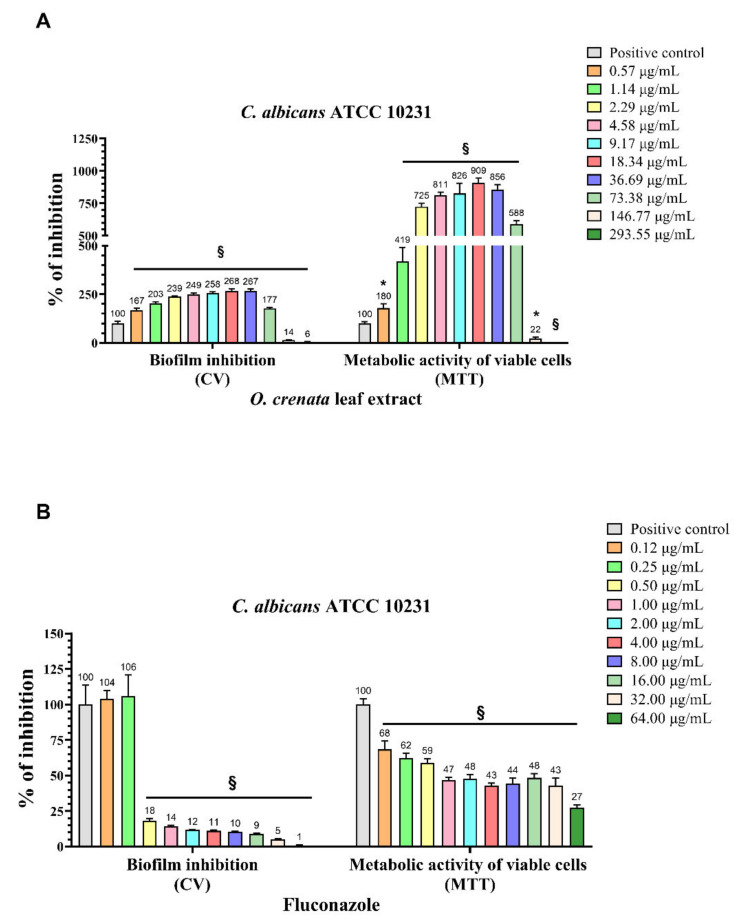
Determination of the effect of *O. crenata* leaf extract and fluconazole (standard drug) on *C. albicans* ATCC 10231 biofilm formation and viability through crystal violet (CV) staining and 3-(4,5-dimethylthiazol-2-yl)-2,5-diphenyltetrazolium bromide (MTT) assay, respectively. *C. albicans* was exposed to increasing concentrations of *O. crenata* extract (**A**), ranging from 0.57 to 293.55 μg/mL, and fluconazole (**B**), ranging from 0.12 to 64.00 μg/mL. In the x-axis, the inhibitory effect of the extract (expressed as percent of positive control) on biofilm formation (CV) and the metabolic activity of viable cells (MTT) of untreated (positive control) and *O. crenata* /fluconazole-treated *C. albicans* are reported. The bars represent the means ± SD of four independent experiments (S.D. = standard deviation). Statistically significant differences were determined using one-way analysis of variance ANOVA and Tukey’s post hoc test. * *p* < 0.05, § *p* < 0.0001 versus positive control.

The ability of OCLE to interfere with the biofilm formation by *C. albicans* and the cell viability of the yeast cells inside the biofilm was evaluated through crystal violet (CV) and MTT assay, respectively (Figure 1A). Results showed a significant increase of both the biofilm formation and cell viability of *C. albicans* following treatment with increasing concentrations of OCLE, ranging from 0.57 to 73.38 µg/mL. These stimulatory effects were counteracted by the two highest concentrations of OCLE (146.77 and 293.55 µg/mL), which instead induced a drastic reduction in both the biofilm formation and yeast cell viability. The anti-biofilm activity of OCLE was compared to the standard antifungal drug fluconazole (Figure 1B). This antimicrobial agent significantly inhibited both the biofilm formation and cell viability of *C. albicans* in a dose-dependent manner.

### 2.3. Germ Tube Assay

**Figure 2:**
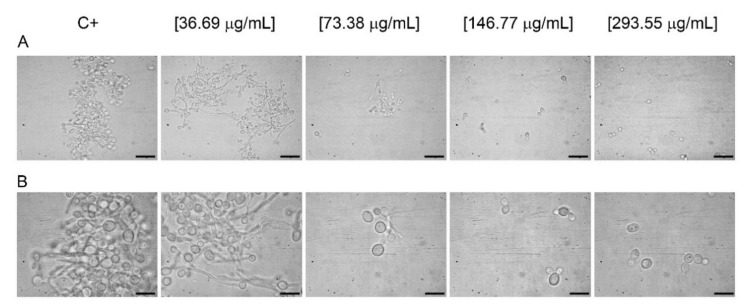
Effect of *O. crenata* leaf extract on the *C. albicans* germ-tube formation and hyphal elongation. *Candida* suspension was incubated at 37 °C for 4 h in the absence (positive control; C+) and presence of four different concentrations of the extract: 36.69, 73.38, 146.77, and 293.55 µg/mL. The microscopic analysis was carried out using the 40× (scale = 50 μm) (**A**) and 100× (scale = 20 μm) (**B**) oil immersion objective plus 10× ocular. Four independent experiments were performed.

To determine the ability of OCLE to inhibit *C. albicans* germ-tube formation, four concentrations of the extract, ranging from 36.69 to 293.55 µg/mL, were tested. The effect was compared to the positive control (inoculum in fetal bovine serum, FBS). The concentration range was selected based on the results of the biofilm assay. Accordingly, the doses of OCLE under 36.69 µg/mL have been considered unable to prevent phenotypic switching of *C. albicans.* The results confirmed this hypothesis, since only the higher concentrations of the extract (146.77 and 293.55 µg/mL) were able to determine a complete inhibition of the germ-tube formation with respect to the positive control. However, it is worth noting that, at the concentration of 73.38 µg/mL, partial inhibition of the yeast-to-hypha transition was observed (Figure 2A,B, Table 2).

### 2.4. Cytotoxicity Assay

**Figure 3:**
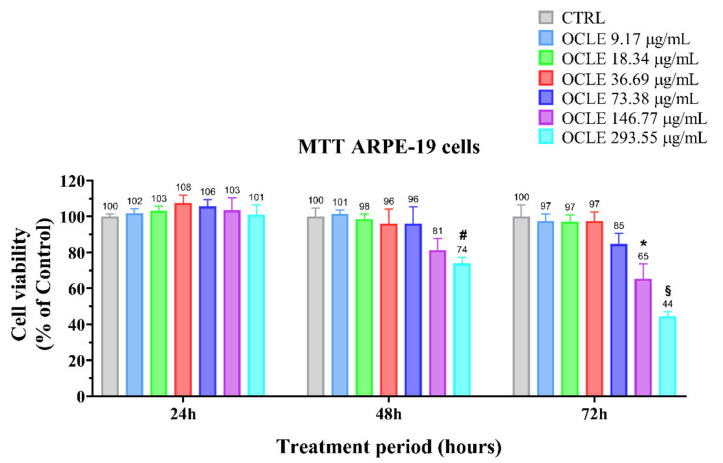
Cell viability of ARPE-19 cells untreated (CTRL) or treated with increasing concentrations of *O. crenata* leaf extract (OCLE), ranging from 9.17 to 293.55 μg/mL, for 24, 48, and 72 h. Values are expressed as mean ± SD of four experiments in triplicate. Significant differences were determined using the two-way Anova test. Significance for pairwise comparison was determined with the Dunnett post hoc test. * *p* < 0.05, # *p* < 0.001, § *p* < 0.0001 versus control at the same incubation time.

The potential cytotoxic effect of OCLE on the non-cancerous human retinal pigment epithelial cell line (ARPE-19) was determined through the MTT assay. The ARPE-19 cell line was cultured in the absence (control) and presence of different concentrations of the natural extract, ranging from 9.17 to 293.55 µg/mL, for 24, 48, and 72 h (Figure 3). The solvent used for the solubilization of the extract (acetone), at a final concentration of 0.05% (*v*/*v*) in the culture medium, did not cause any change in cell viability (data not shown). The treatment with OCLE at 24 h did not affect ARPE-19 cell viability, at all tested concentrations. However, after 48 h, the exposition of ARPE-19 cells to the highest concentration (293.55 µg/mL) of OCLE induced a reduction of about 30% in cell viability with respect to untreated cells (control). This effect became more evident at 72 h, at which a significant reduction of ARPE-19 cell viability was observed already at 146.77 µg/mL.

### 2.5. Wound Healing Assay

**Figure 4:**
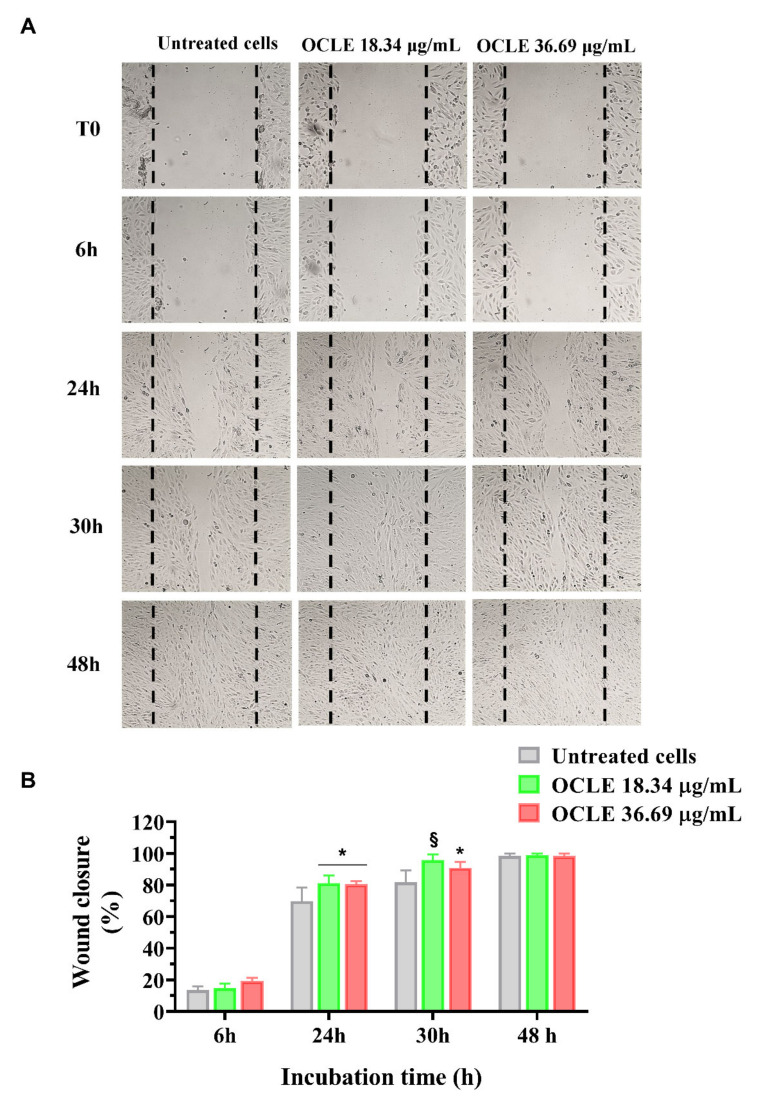
Re-epithelizing effect of *O. crenata* leaf extract on ARPE-19 cells. ARPE-19 cells were cultured in the absence (untreated) or in the presence of 18.34 and 36.69 µg/mL of the extract. (**A**) Representative images of ARPE-19 reparative migration at different time points (6, 24, 30, and 48 h) after the creation of the wound (time 0; T0). (**B**) Quantitative analysis of reparative migration of ARPE-19 cells grown in the absence (untreated) or the presence of 18.34 and 36.69 µg/mL of OCLE. Results are expressed as the percent of wound closure vs. incubation time. Data are reported as the mean ± SD of three independent experiments performed in triplicate. Significant differences were determined using the two-way Anova test. Significance for pairwise comparison was determined with the Dunnett post hoc test. * *p* < 0.05, § *p* < 0.0001 versus control at the same incubation time. OCLE: *O. crenata* leaf extract. CTRL: control.

To evaluate the potential stimulatory effect of OCLE on ARPE-19 cell migration, we performed the wound healing assay. Cell migration was monitored for 6, 24, 30, and 48 h following the scratch and the representative images of each time point are shown in Figure 4A. ARPE-19 cells were treated with three different concentrations of OCLE ranging from 18.34 to 73.38 μg/mL, which were subtoxic by the MTT assay. ARPE-19 cells stimulated with 18.34 and 36.69 μg/mL of OCLE showed faster reparative migration compared to the untreated cells (control), particularly at the lowest tested concentration. Indeed, cells treated with 18.34 μg/mL completely closed off the wound after 30 h of incubation (Figure 4A). Otherwise, OCLE at the concentration of 73.38 μg/mL did not determine any effect on ARPE-19 migration compared to the untreated cells (data not shown). Quantitative analysis of ARPE-19 migration reflects the results observed in Figure 4A. After 24 h, the treatment with both 18.34 and 36.69 μg/mL of OCLE was able to induce a significant increment of ARPE-19 cell migration compared to the untreated cells. However, at 30 h, the lower concentration of OCLE (18.34 μg/mL) was more efficient in promoting the re-epithelization of the wound with respect to 36.69 μg/mL, allowing ARPE-19 cells to reach 100% of wound closure (Figure 4B).

### 2.6. Effect on the Adhesion of Candida on the Human Retinal Pigment Epithelial Cell Line (ARPE-19)

**Figure 5:**
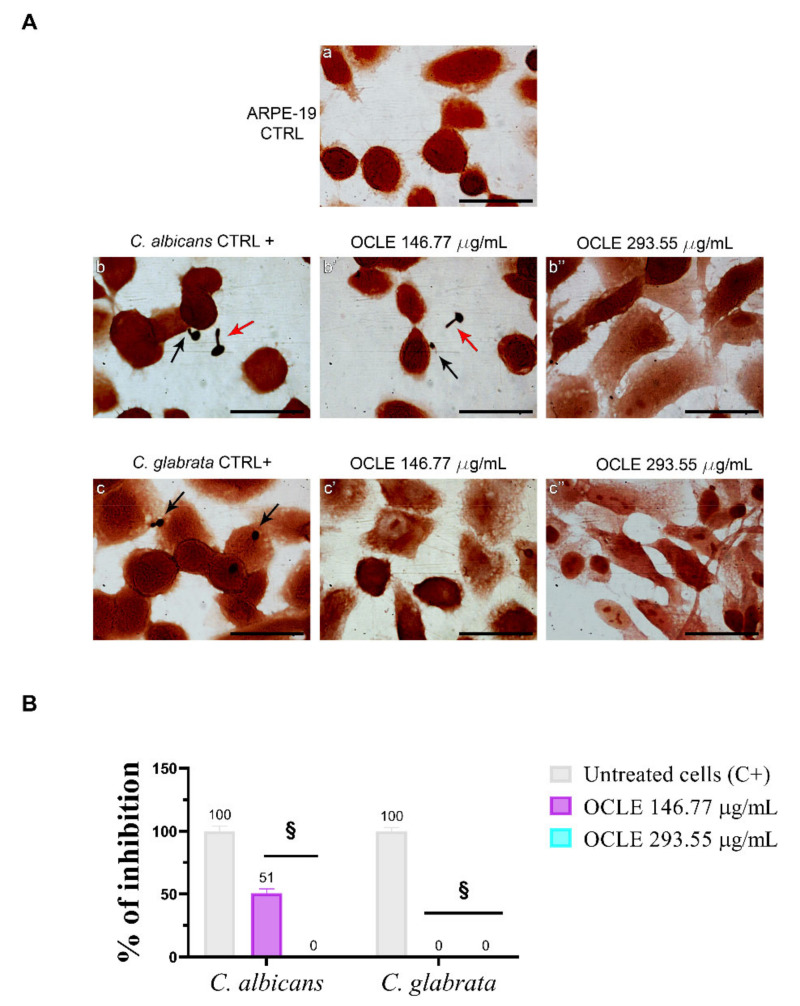
Effect of *O. crenata* leaf extract (OCLE) on *Candida* adhesion to ARPE-19 cells. (**A**) Adherent cells were observed by light microscopy at a magnification of 100× plus 10× ocular (scale = 50 μm). Yeast adhesion, in the different experimental conditions, was compared to the negative control. (a): uninfected ARPE-19 cells; (b): ARPE-19 cells infected with *C. albicans* (positive control); (b’): ARPE-19 cells infected with *C. albicans* and simultaneously treated with 146.77 µg/mL of OCLE; (b’’): ARPE-19 cells infected with *C. albicans* and simultaneously treated 293.55 µg/mL of OCLE; (c): ARPE-19 cells infected with *C. glabrata* (positive control); (c’): ARPE-19 cells infected with *C. glabrata* and simultaneously treated with 146.77 µg/mL of OCLE; (c’’): ARPE-19 cells infected with *C. glabrata* in and simultaneously treated with 293.55 µg/mL of OCLE. Black arrow: adherent *Candida* cells; red arrow: non-adherent *Candida* cells. (**B**) Quantitative analysis of *C. albicans* and *C. glabrata* adhesion on ARPE-19 cells following treatment with OCLE. ARPE-19 cells infected with *C. albicans* or *C. glabrata* were cultured in the absence (untreated cells; C+) and the presence of 146.77 or 293.55 µg/mL of the extract. Cell adhesion assay was performed for 1 h of incubation at 37 °C. Values are expressed as mean ± SD of three experiments considering 10 microscopic fields. Significant differences were determined using the two-way Anova test. Significance for pairwise comparison was determined with the Dunnett post hoc test. § *p* < 0.0001 versus positive control.

After the treatment with two different concentrations of OCLE (146.77 and 293.55 µg/mL), the glass coverslips were fixed and Gram-stained to count adherent *Candida* cells. These concentrations were chosen based on the phenotype switching experiment, in which the most effective concentrations able to inhibit morphological change of *C. albicans* were 146.77 and 293.55 µg/mL. The number of yeasts adhering to 100 ARPE-19 cells was determined by light microscopy at a magnification of 100× plus 10× ocular. The adhesion capacity of *C. glabrata* ATCC 2001 (50 cells/100 ARPE-19 cells) was highest with respect to *C. albicans* ATCC 10231 (31 cells/100 ARPE-19 cells). OCLE, at the concentration of 146.77 μg/mL, reduced the adhesion of *C. albicans* by 49% compared to the untreated cells and, at 293.55 μg/mL, totally prevented the interactions. Notably, the two tested concentrations of OCLE were able to inhibit the adhesion of *C. glabrata* to ARPE-19 cells (Figure 5A,B, Table 3).

### 2.7. Determination of Total Phenols Content of O. crenata Leaf Extract

The total phenols content was determined through the Folin–Ciocalteau assay, by comparing the absorbance of different concentrations of OCLE to the gallic acid standard solutions. Quantitative analysis indicated that with increasing concentration of the extract, the total phenols content had a higher value. The obtained values ranged from 186 ± 1.23 to 209 ± 3.79 μM gallic acid equivalents/L (Table 4).

### 2.8. Chemical Analysis

To correlate the antifungal effect of OCLE with one or more bioactive molecules present in the extract, we performed UPLC-Ms/Ms. The compounds and the related chemical structures are reported in Table 5. Interestingly, some molecules detected in the extract, such as acteoside, apigenin, and luteolin, were found to have anti-candidal, anti-biofilm, and anti-hyphal forming activities. Interestingly, besides the above-mentioned compounds, salidroside showed a protective activity on retinal cells. All this evidence supports our findings, revealing that the antifungal activity and the re-epithelizing effect on ARPE-19 cells of OCLE could be mediated by these bioactive molecules. The tandem mass spectra and chromatograms of OCLE are available in the Supplementary Materials (Figures S1–S12).

## 3. Discussion

The genus *Candida* includes numerous species, most of which are part of the human microbiome. However, among these commensals, *C. albicans* and *C. glabrata* are known for their ability to cause infections in humans, particularly in immunocompromised patients [59,60]. In this regard, it is worth highlighting that the ongoing pandemic by SARS-CoV-2 dramatically increased the number of hospitalizations. This condition further contributed to the development of *Candida* opportunistic infections, exacerbating the consequences of fungal infections in a context characterized by highly susceptible patients [61,62,63].

*Candida*-related endophthalmitis and chorioretinitis are serious complications often arising from disseminated candidemia. In these cases, a timely and effective therapy is essential to avoid the complete visual loss of the affected patients. The antifungal drugs used for the treatment of *Candida*-related infections can be distinguished into four classes, including the azoles, polyenes, echinocandins, and pyrimidine analogue flucytosine. Among these agents, azoles and echinocandins are considered elective therapy for the treatment of systemic candidiasis [64]. However, in the last decades, the efficacy of antifungal drugs has been highly challenged by the spread of antimicrobial-resistant fungal pathogens [24,65]. Specifically, azole resistance has been proved in HIV-infected patients [66,67] and in those who undergo antifungal prophylaxis [68]. Moreover, it has been reported that chronic exposition to echinocandin exerts selective pressure on *C. glabrata*, promoting the acquisition of mutations that weaken the efficacy of the antifungal drug [69]. The emergence of resistant strains to the most used antifungal drugs constitutes a considerable public health threat.

In this scenario, the exigence of new and efficient antifungal agents that allow the prevention of antimicrobial resistance-related therapeutic failure is becoming increasingly urgent. The research and development of novel drugs can be undoubtedly favored by the study of phytoextracts [70], since they contain a pool of bioactive compounds exerting a wide range of biological activities, including antimicrobial ones [71,72,73]. Specifically, it has been shown that phenolic compounds, isolated from natural sources, exhibited anticandidal activity [73]. However, although the scientific interest for plant-derived extracts has increased in the last few years, the knowledge on the biological properties of numerous officinal plants remains limited.

Concerning this, *O. crenata*, a parasitic plant particularly widespread in the Mediterranean area including Sicily, is one of the still poorly studied and characterized plants, especially regarding the biological role of its extracts [36]. However, in our previous paper, we partially filled this knowledge gap, proving the ability of the acetonic leaf extract to counteract the growth of several clinically relevant bacterial and *Candida* spp. strains [35]. The obtained results led us to deepen the antifungal and anti-invasive activity of OCLE on both *C. albicans* and *C. glabrata*.

Accordingly, in the present study, we demonstrated that OCLE efficiently inhibited the growth of both *C. albicans* and *C. glabrata*, with MIC values of 146.77 and 73.38 µg/mL, respectively. However, this extract showed a fungistatic action, since yeast cells, exposed to OCLE, undergo cell growth arrest but not cell death. On the other hand, it is important to note that a limited range of concentrations (between 0.57 and 273.55 µg/mL) was tested and, therefore, it is possible to hypothesize that a fungicidal action could be induced by higher doses of the extract. Nevertheless, according to literature data, a natural extract possesses a significant antimicrobial activity if MIC values are below 100 µg/mL and moderate when the MIC values range between 100 and 625 µg/mL [74,75,76]. Based on these criteria, our extract is endowed with a noteworthy antifungal activity.

Besides the effect of the extract on fungal growth, we proposed evaluation of the anti-invasive activity of OCLE against two fungal pathogens. It is well established that biofilm is a survival strategy used by microorganisms to resist antimicrobials and immune attacks. The production of this polymeric matrix is one of the virulence mechanisms adopted by *C. albicans* and *C. glabrata* to persist in the host. Indeed, both organisms are able to form biofilm on different types of indwelling medical devices, such as urinary catheters, prosthetic heart valves, and dentures [77,78,79]. The permanence of yeast cells inside the biofilm (sessile cells) exposes patients to recurrent infections. Indeed, favorable conditions promote the release of planktonic cells from the biofilm, which, in the absence of an efficient immune system, can reach the bloodstream, thus provoking systemic candidiasis [80]. Although *C. glabrata* normally forms the biofilm, we found that this strain, in our experimental conditions, lacks this capacity. However, this evidence is consistent with the study by Alnuaimi et al., which revealed that the standard strain *C. glabrata* ATCC 2001 is one of the most scarce biofilm-producing strains [81]. Therefore, we tested the potential inhibitory effect of OCLE on biofilm formation only on *C. albicans*. It is worth noting that the treatment of *C. albicans* with increasing concentrations of OCLE, ranging from 0.57 to 293.55 µg/mL, produced a double effect on biofilm formation. Specifically, *C. albicans* exposed to lower OCLE concentrations, between 0.57 and 73.38 µg/mL, showed a significant increment in biofilm production and consequently also on yeast cell viability. Conversely, the two higher concentrations of 146.77 and 293.55 µg/mL caused a drastic reduction of both biofilm formation and cell viability. This response could be the result of the hormetic mechanism, which is characterized by a biphasic dose–response deriving from a low dose stimulation and a high dose inhibition [82,83,84]. The hormesis can be viewed as an adaptative response to stress conditions, induced by chemical and physical agents interfering with cell physiology [85]. This phenomenon was firstly described in 1943 by Southam and Ehrlich, who observed a stimulatory effect of red cedar tree extract on the growth and metabolism of different fungal strains [86]. Based on this concept, we can hypothesize that lower doses of the extract trigger a compensatory response as a result of an adaptative mechanism against an alteration in cellular homeostasis. However, this positive reaction is dose dependent. Accordingly, higher doses of OCLE showed an inhibitory effect on biofilm formation and cell viability. Several in vitro and in vivo studies reported that the extract of many herbs induces a hormetic response [85,87,88,89,90]. For this reason, hormesis should be taken into consideration to establish the dose of herbal medicine that allows a health benefit effect to be obtained [85].

Another important feature associated with *Candida* invasiveness is hypha formation. *C. albicans*, in response to environmental changes, is able to switch from a yeast (spherical) to hyphal (filamentous) form. This reversible phenomenon is commonly defined as “plasticity”. The yeast-to-hypha transition allows the microorganism to change from a commensal to opportunistic pathogen, also responsible for severe infections [91]. In our study, the microscopic observation of *C. albicans* after the treatment with OCLE revealed a block in hyphal elongation. This effect was notable at the concentrations of 146.77 and 293.55 µg/mL, respectively. Raut et al. demonstrated that the benzenoid vanillin, at the concentration of 500 µg/mL, was able to inhibit the switch of *C. albicans* from yeast to hypha [92,93]. In particular, vanillin is a metabolite derived from phenylpropanoids following the loss of two carbons of the side chain [94]. It is worth noting that this compound was isolated from *Orobanche speciosa* by gas chromatography-mass spectrometry [95] and it seems to be an inductor of haustoria formation [96]. Haustoria are organs produced by parasitic plants that allow the invasion of host root tissues [96].

*Candida* ocular infection is a serious complication of candidemia, a systemic infection that frequently occurs in immunocompromised patients [13,97]. Considering the devastating effects of ocular candidiasis, we proposed an investigation of the possible stimulatory action of OCLE on ARPE-19 cell migration. For this purpose, we first evaluated the possible cytotoxic effect of OCLE on this cell line. The results showed that the treatment for 24 h with OCLE did not produce any change in ARPE-19 cell viability. However, the prolonged exposition to 146.77 and 293.55 µg/mL of the extract significantly affected ARPE-19 cell viability, mainly at 72 h. Although OCLE, at the concentrations mentioned before, efficiently counteracted the growth, biofilm formation and cell viability of *Candida* strains, these effects were obtained after 48 h of incubation. Therefore, at this time point, only the highest dose of OCLE (293.55 µg/mL) induced a slight, even if significant, decrease in cell viability. The cytotoxic effect induced by OCLE on ARPE-19 cells is due to a higher susceptibility of animal cells compared to yeast cells to the action of xenobiotic agents [98]. Therefore, this aspect should be noted for future possible pharmacological applications of this natural extract.

Regarding the potential re-epithelizing effect of the extract, the treatment of ARPE-19 cells with 18.34 and 36.69 µg/mL of OCLE induced a significant enhancement of ARPE-19 cell migration compared to untreated cells. The effect was more evident on ARPE-19 cells treated with 18.34 µg/mL, which were able to repair the wound already at 30 h following the scratch. The stimulatory effect of OCLE on cell migration decreases in a dose-dependent manner. Indeed, at the highest tested concentration (73.38 µg/mL) of the extract, ARPE-19 cell migration was similar to that of unstimulated cells (data not shown). It is well established that hormesis modulates different biological processes, including cell migration [99]. In this regard, an interesting paper by Demirovic and Rattan reported that curcumin, a phenolic compound present in *Curcuma longa*, induces a biphasic response on normal adult skin fibroblast cell migration, showing a stimulatory activity at low doses and inhibitory activity at higher doses [100]. Considering that our extract is rich in polyphenols, we can speculate that one or more phenolic compounds contained in OCLE could mediate the hormetic response underlying ARPE-19 cell migration.

To further elucidate the anti-invasive effects of our extract, we thought of studying the adhesion of *Candida* species on ARPE-19 cells. Indeed, adhesiveness to cells represents an essential stage in the pathogenesis of infection and the formation of microbial biofilm [5]. A significant reduction (*p* < 0.0001) of *C. albicans* and *C. glabrata* adhesion to ARPE-19 cells was observed in the presence of OCLE. The extract in general and more specifically its phenolic constituents could have altered the hydrophobicity of the cell membrane, making it more hydrophilic and causing detachment from the host cell [101,102]. Besides, the functional group attached to the phenyl ring plays a crucial role in inhibiting the yeast–cell interaction [103]. Raut et al. reported that phenylpropanoids of plant origin, chemical compounds isolated also from *O. crenata* [36], are able to prevent the growth, adhesion, yeast-to-hypha transition, and biofilm formation of *C. albicans* [93]. The anti-adhesive and anti-biofilm effects of the phenylpropanoid eugenol were also demonstrated against non-*Candida albicans* species [104].

Concerning the quantitative chemical analysis of OCLE, the total phenols content ranged from 186 ± 1.23 to 209 ± 3.79 μM gallic acid equivalents/L. Therefore, the solvent used in the extraction process (acetone) allowed us to obtain a high concentration of these compounds. Indeed, it is well known that acetone represents one of the most suitable solvents for recovering phenols from vegetable matrices [105]. Furthermore, our findings are corroborated by a recent paper by Attia et al. In this study, the authors evaluated the biological activities and chemical composition of three parasitic plants harvested in Tunisia, including *O. crenata*. The natural extracts were obtained by macerating plants’ aerial parts in five different solvents: hexane, methanol, ethyl acetate, acetone, and water. Interestingly, the acetonic extract of *O. crenata* aerial parts presented the highest content of phenolic compounds [106]. Furthermore, this result is consistent with the UPLC-Ms/Ms analysis, which substantially confirmed the presence of several phenolic compounds (Table 5). Interestingly, previously published papers demonstrated an anti-candidal, anti-biofilm, anti-hyphal forming, and cytoprotective effect on retinal cells of some of the UPLC-detected molecules, including acteoside [37,38,39,40,41,42], apigenin [43,44,45,46,47,48,49], luteolin [46,50,51,52,53,54], and salidroside [55,56,57,58]. Since all these biological activities reflect the obtained results, it is possible to hypothesize that the antifungal and re-epithelizing effects of OCLE could be mediated by these chemical agents.

## 4. Materials and Methods

### 4.1. Chemicals

All the solvents and chemical compounds were of reagent grade and purchased from Sigma-Aldrich (Milan, Italy).

### 4.2. Plant Material and Preparation of the Extract

**Figure 6:**
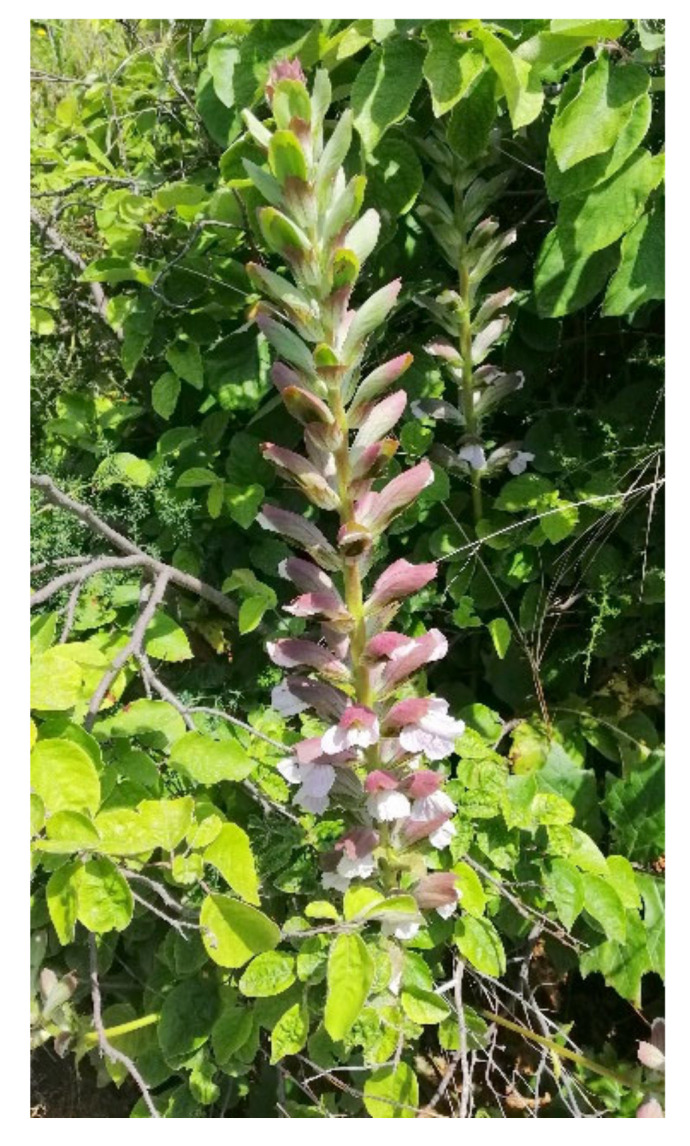
*Orobanche crenata* Forssk.

*O. crenata* (Figure 6) was collected on May 2019 in Modica (Ragusa, Italy; Latitude 36°51′48.93″ N, Longitude 14°45′53.69″ E, Altitude 382 m). A voucher specimen (n° 35/04) was deposited in the herbarium of the Department of Drug and Health Sciences, University of Catania. The extract was obtained by macerating 10 g of ground leaves in 200 mL of acetone at room temperature (RT) for 72 h. After filtration through a Whatman^®^ Grade 1 filter paper (Whatman, UK), the extract was evaporated at 40 °C by a rotatory evaporator (Stuart RE300) under reduced pressure, obtaining 0.58710 g (±0.1 mg) of dry extract.

### 4.3. Microorganisms

In the present study, two American Type Culture Collection (ATCC) strains were used: *C. albicans* ATCC 10231 and *C. glabrata* ATCC 2001. The fungal strains were purchased from LGC Limited (Teddington, Middlesex, UK).

### 4.4. Antifungal Susceptibility Test

The antifungal activity of the natural extract was evaluated according to the standard procedures of the Clinical and Laboratory Standards Institute (CLSI) M27-A3 [107]. The strains were grown on Sabouraud dextrose agar plates (SDA) (Oxoid, Milan, Italy) at 35 °C for 48 h. The cell suspensions were prepared in 5 mL of 0.145 M sterile saline solution and adjusted to 0.5 McFarland scale (1.5 × 10^8^ Colony Forming Units (CFUs)/mL) by a spectrophotometer (Bio-Tek Synergy HT Microplate Reader, Bio-Tek Instruments, Winooski, USA) at λ = 530 nm. For the antifungal susceptibility test, the culture medium bicarbonate-free Roswell Park Memorial Institute (RPMI) 1640 with l-glutamine, buffered to pH 7.0 with 0.165 M morpholinepropanesulfonic acid (Sigma-Aldrich, Milan, Italy), was used. OCLE was diluted in the 1:100 ratio in RPMI 1640 medium. Ten concentrations ranging from 0.57 to 293.55 µg/mL were obtained in sterile 96 U-well microplates (Corning, New York, NY, USA). The antifungal agent fluconazole, in concentrations ranging from 0.125 to 64.00 µg/mL, was used as the positive control. The final concentration of the inoculum was from 5 × 10^2^ to 2.5 × 10^3^ cells/mL per well.

To determine MFC_50_, 100 µL of sample were removed from the wells of the MIC_50_ and subcultured in SDA plates. After incubation at 35 °C for 48 h, the CFUs were counted. Four independent experiments were performed.

### 4.5. In Vitro Biofilm Formation and Inhibition Assay

The biofilm formation and inhibition assay were performed according to the method of Melo et al. [108] with some modifications. For the determination of biofilm formation, 200 µL of *Candida* strains suspensions 1.0 × 10^7^ cells/mL in RPMI 1640 were added in flat-bottomed 96-well microtiter plates (Corning, New York, NY, USA). The microplates were incubated for 48 h at 37 °C to allow the growth of the biofilm.

For the determination of the anti-biofilm activity of OCLE, 100 µL of cell suspensions (1.0 × 10^7^ cells/mL) in RPMI 1640 were inoculated in the flat-bottomed 96-well microplate. Afterwards, 100 µL of the serial dilutions of the extract, in concentrations ranging from 1.14 to 587.10 µg/mL, were added to the microplate After incubation for 48 h at 37 °C, the wells were discharged and washed twice with 200 µL of phosphate-buffered saline (PBS). The biofilm was stained with 200 µL of 0.4% (*v*/*v*) aqueous CV solution (Merck, Damm, Germany) for 45 min. Subsequently, the wells were discharged and washed twice with 200 µL of PBS. The microplates were air-dried and the biofilm-bound CV was dissolved with 200 µL of 95% (*v*/*v*) ethanol. Absorbance was measured through the spectrophotometer at λ = 595 nm. Four independent experiments were performed.

### 4.6. Determination of Fungal Viability

The viability of fungal strains inside biofilm was determined by the MTT assay. The method of Ansari et al. with some modifications was used [109]. After the MBIC_50_ assay, the wells were discharged and washed twice with 200 µL of PBS. Then, 0.5 mg/mL of MTT solution in PBS was added to the flat-bottomed 96-well microplate and incubated at 37 °C for 5 h. The purple formazan inside biofilms was dissolved with 200 µL of dimethyl sulfoxide (DMSO). Afterwards, the microplates were incubated for 20 min, with agitation, in the dark, at RT. Metabolically active cells were able to metabolize the yellow tetrazole into insoluble purple formazan. The O.D. was determined through the spectrophotometer at λ = 570 nm. The metabolic activity was determined by comparing the O.D. of treated cells with the drug-free control. Four independent experiments were performed.

### 4.7. Germ Tube Assay

The effect of OCLE on *C. albicans* ATCC 10231 tube formation was studied through the method of Bernardes et al., with slight modifications [110]. *C. albicans* germ-tube formation was induced in Sabouraud Dextrose Broth (SDB) (Oxoid, Milan, Italy) with 10% (*v*/*v*) of fetal bovine serum (FBS) (Sigma-Aldrich, Milan, Italy). Then, 100 µL of fungal suspensions 1.0 × 10^7^ cells/mL were incubated in tubes containing 2 mL of SDB, 10% of FBS (*v*/*v*), and four different concentrations of OCLE (36.69, 73.38, 146.77, and 293.55 µg/mL). After 4 h of incubation at 37 °C, aliquots were taken and observed microscopically. Microscopic analysis was performed using the 40× and 100× oil immersion objective plus 10× ocular (Leica DMRB Fluorescence Microscope). Digital images were acquired through a computer-assisted digital camera (Leica DFC 320, 3.3 Megapixel; Software: Leica Application Suite 2.8.1). Four independent experiments were performed and the inoculum with FBS served as the positive control.

### 4.8. Cell Culture

The human retinal pigment epithelial cell line (ARPE-19) ATCC^®^ CRL-2302^TM^ was purchased from LGC Limited (Teddington, Middlesex, United Kingdom). The cells were maintained in a mixture (1:1 ratio) of Dulbecco’s modified Eagles medium and Ham’s F12 medium with HEPES buffer (DMEM/F-12-HEPES; Gibco^TM^, catalog number 11330032; Thermo Fisher Scientific, Inc.) supplemented with 20% *v*/*v* of heat-inactivated FBS (Sigma-Aldrich, Milan, Italy) and 1% (*v*/*v*) of penicillin/streptomycin (Sigma-Aldrich, Milan, Italy) and incubated at 37 °C in a humidified incubator with 5% CO_2_. The cells were passaged once a week following trypsinization and replaced with a new medium twice weekly. 

The ARPE-19 cells, at passage 8, were cultured in the presence or absence (control) of increasing concentrations of OCLE, ranging from 10 to 120 µg/mL, for 24, 48, and 72 h. The treatments were performed using DMEM/F-12-HEPES supplemented with 1% (*v*/*v*) of FBS (starvation conditions), to minimize cell proliferation induced by the medium [111]. The final concentration of acetone (solvent used for extract solubilization) in the culture medium was less than 0.01% (*v*/*v*). This low concentration excludes any possible effect of the vehicle on treated cells [112].

### 4.9. Cytotoxicity Assay

To evaluate the possible cytotoxic effect of the extract on ARPE-19 cells, the MTT assay was used, as previously reported [113]. Briefly, cells were seeded in 96-well microplates, at a density of 1.5 × 10^4^ cells per well and incubated overnight at 37 °C before the treatments. Following this, the cells were exposed to scalar concentrations of OCLE (10, 20, 40, 60, 120 μg/mL) for 24, 48, and 72 h. Afterwards, 10 μL of MTT reagent (5 mg/mL) were added to each well and the cells were incubated for 3 h at 37 °C. The formazan crystals were solubilized with 100 μL of DMSO and the microplates were shaken for 10 min. The absorbance was measured at λ = 570 nm. Results were expressed as mean ± SD of four experiments in triplicate.

### 4.10. Wound Healing Assay

To verify the ability of OCLE to promote the repair of retinal damage induced by *Candida* infection, we performed a wound healing assay, as previously described [114,115]. Briefly, ARPE-19 cells were seeded into 12-well plates. Upon reaching the confluence, the cell monolayer was wounded using a 200 μL pipette sterile tip. After the scratch, the wells were washed three times to remove cell debris and incubated with 1% *v*/*v* FBS medium alone (control; CTRL) or in combination with OCLE at different concentrations (18.34, 36.69, and 73.38 μg/mL). Migration of ARPE-19 was monitored for 6, 24, 30, and 48 h after the scratch (Time 0; T0).

The percentage of wound closure was evaluated by applying the following formula:

Percentage migration = (A_t0_ − A_migration_)/A_t0_ × 100%

where A_t0_ is the wound area measured at T0 and A_migration_ is that measured at a precise time point, as previously reported [116]. An inverted Leica DM IRB microscope equipped with a CCD camera (Leica Microsystems, Inc., Bucharest, Romania) was used to monitor ARPE-19 cell migration. Results were expressed as the mean ± SD of three independent experiments performed in triplicate.

### 4.11. Adhesion Assay on Human Retinal Pigment Epithelial Cell Line (ARPE-19)

The adhesion assay was performed according to the protocol of Dalle et al. [117], with some modification. ARPE-19 cells were grown in a 12-well microplate (Corning, New York, NY, USA) containing 12 mm glass coverslips (Thermo Scientific Menzel) at a density of 1.5 × 10^4^ cells/well. Cell monolayers were inoculated with a *C. albicans* suspension 10^3^ cells/mL and incubated at 37 °C for 1 h. The wells were rinsed three times with PBS to remove non-adherent cells and fixed with 2% (*v*/*v*) glutaraldehyde for 10 min. The number of adherent cells was determined through the Gram-staining procedure [118] and observed with an optical microscope at 100× oil immersion magnification plus 10× ocular. The adhesion assay was repeated three times under the same conditions.

### 4.12. Total Phenolic Content

The total phenolic content was determined through the Folin–Ciocalteau method, as previously described [31]. The standard curve was constructed by using known concentrations of gallic acid. The diluted samples of OCLE (0.1 mL) and gallic acid (0.1 mL) were transferred in 15 mL test tubes. Afterward, the Folin–Ciocalteau reagent (3.0 mL, 0.2 N) was added to each tube and vortexed. After 1 min, 2 mL of 9.0% (*w*/*v*) Na_2_CO_3_ in water were added and absorbance was measured at λ = 765 nm. The total phenolic content was determined by comparing the absorbance of the natural extract with that of the acid gallic. The experiment was performed in triplicate.

### 4.13. Chemical Analysis

The chemical composition of OCLE was determined through UPLC-Ms/Ms (Perkin-Elmer FX 10/AB SCIEX API 2000TM). The separation was performed by using acetonitrile-water 90:10 (*v*/*v*) mixture with 0.1% (*v*/*v*) acetic acid as the mobile phase. The elution rate was 200 µL/min for 120 min. The analysis was accomplished by a C18 column (Phenomenex Luna, 2.6 µm, 100 × 2.1 mm) and a total volume of 10 µL was injected. The UV detector was monitored at an absorbance of 220 nm. Electrospray Ionisation-Ms/Ms was used in positive and negative modes, for detailed spectrum analysis. The settings of the equipment are shown below: Ion Spray Voltage (IS) 5500/-4500, Curtain gas 30, Ion Source Gas1 (GS1) 30.0, Ion Source Gas2 (GS2) 60.0, Declustering Potential (DP) 150.0, Focusing Potential (FP) 400.0 Entrance Potential (EP) 10.0, and Temperature (TEM) 350 °C.

### 4.14. Statistical Analysis

Data are expressed as the mean ± standard deviation (±SD) of three independent experiments, performed in triplicate. We evaluated the statistical significance of these data by applying one-way Anova or two-way Anova as described in figure legends.

## 5. Conclusions

In light of these considerations, we can conclude that *O. crenata* represents a rich source of compounds able to modulate different biological functions. The ability of *O. crenata* to inhibit the growth and invasiveness of two clinically relevant *Candida* species, *C. albicans* and *C. glabrata*, allowed us to widen the current knowledge regarding the antimicrobial properties of this parasitic plant. Besides, the re-epithelizing effect on human retinal pigment epithelial cells represents an interesting biological aspect that deserves to be further explored. All these effects could be mediated by the bioactive molecules highlighted by UPLC-Ms-Ms analysis. Therefore, our findings encourage further studies aimed to explore the mechanism of action by which these molecules act.

## Figures and Tables

**Table 1:** MICs and MFCs values (µg/mL) of *O. crenata* against *Candida* strains.

Fungal Strains	OCLE ^1^		Fluconazole	
	MFC_50_ ^2^	MIC_50_ ^3^	MIC_50_	I.C. ^4^
*Candida albicans* ATCC 10231	>293.55	146.77	16.00	S-DD
*Candida glabrata* ATCC 2001	>293.55	73.38	16.00	S-DD

^1^ OCLE: *Orobanche crenata* leaves extract; ^2^ MFC_50_: Minimal Fungicidal Concentration 50; ^3^ MIC_50_: Minimal Inhibitory Concentration 50; ^4^ I.C.: Interpretive criteria for fluconazole (CLSI M27-A3): ≤8.00 µg/mL Susceptible (S); 16.00–32.00 µg/mL Susceptible-dose-dependent (S-DD); ≥64.00 µg/mL Resistant (R); Fluconazole was used as a positive reference standard. Results are expressed as the mean of four experiments.

**Table 2:** Germ-tube inhibitory activity of *O. crenata* leaf extract.

Fungal Strain	OCLE ^1^ (µg/mL)				
	C+ ^2^	36.69	73.38	146.77	293.55
*Candida albicans* ATCC 10231	-	-	+	++	++

^1^ OCLE: *Orobanche crenata* leaf extract; ^2^ C+: positive control (inoculum with FBS); -: no inhibition; +: partial inhibition; ++: complete inhibition.

**Table 3:** Counts of yeasts that adhered to ARPE-19 cells in the presence of *O. crenata* leaf extract.

Fungal Strains	Treatment	Number of Adhered Yeasts/100 Cells (% Adherence)	*p*-Value
*Candida albicans* ATCC 10231	CTRL+ ^1^	31/100 (100%)	-
	OCLE ^2^ 146.77 µg/mL	16/100 (51%)	*p* < 0.0001
	OCLE 293.55 µg/mL	0/100 (0%)	*p* < 0.0001
*Candida glabrata* ATCC 2001	CTRL+	50/100 (100%)	-
	OCLE 146.77 µg/mL	0/100 (0%)	*p* < 0.0001
	OCLE 293.55 µg/mL	0/100 (0%)	*p* < 0.0001

^1^ CTRL+: positive control (ARPE-19 cells plus fungal strains); ^2^ OCLE: *Orobanche crenata* leaf extract. The adhesion assay was repeated three times under the same conditions (*n* = 3).

**Table 4:** Total phenols content of *O. crenata* leaf extract.

	OCLE ^1^ (µg/mL)					
	9.17	18.34	36.69	73.38	146.77	293.55
Total phenols content ^2^	186 ± 1.23	188 ± 1.89	194 ± 1.72	199 ± 0.44	203 ± 1.59	209 ± 3.79

^1^ OCLE: *Orobanche crenata* leaf extract; ^2^ Total phenols content is expressed in μM gallic acid equivalents/L. Data are expressed as mean ± SD (*n* = 3).

**Table 5:** Chemical compounds identified from *O. crenata* leaf extract through UPLC-Ms/Ms and related biological activities (anti-candidal, anti-biofilm, anti-hyphal, and cytoprotective effect on retinal cells).

Chemical Name	Chemical Structure	m/z (g/mol)	Peak (Polarity)	RT * (min)	Biological Activities
3-O-acetylepisamarcandin	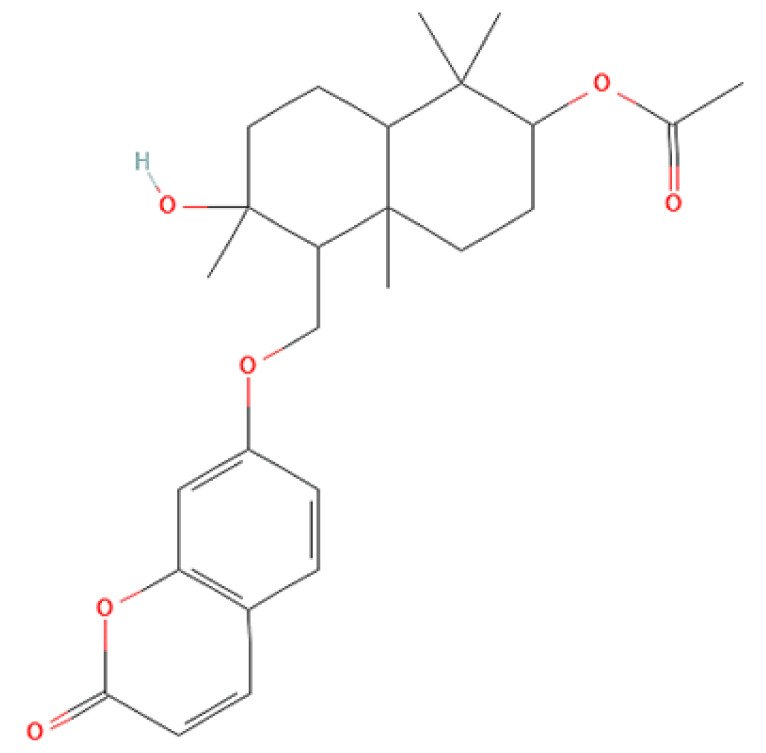	442.236	443.24 (+) 441.22 (−)	38.62	n.a.
Acteoside	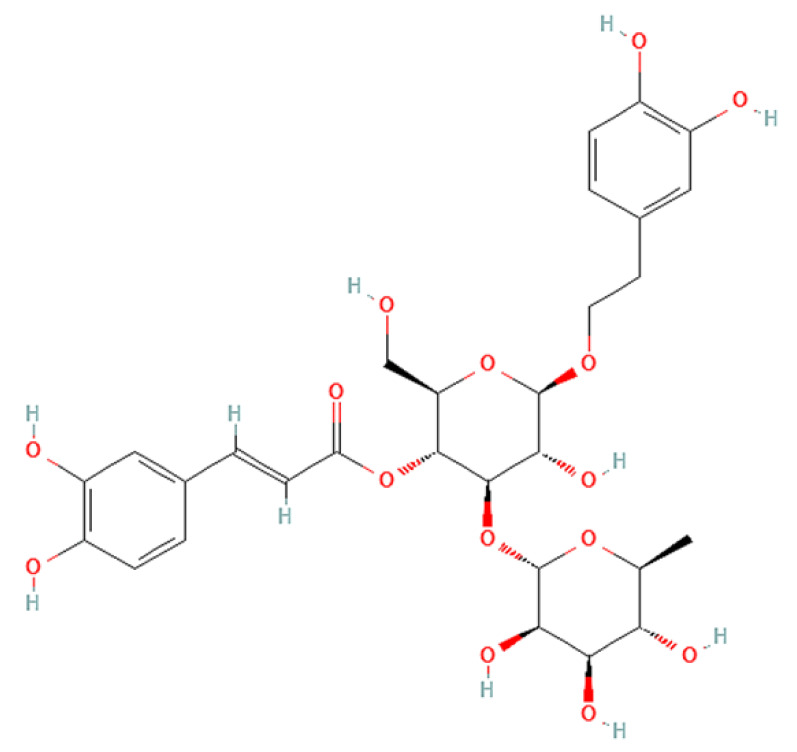	624.205	625.21 (+) 623.19 (−)	23.23	Anti-candidal [37,38,39] Anti-biofilm [39] Cytoprotective (retinal cells) [40,41,42]
Apigenin	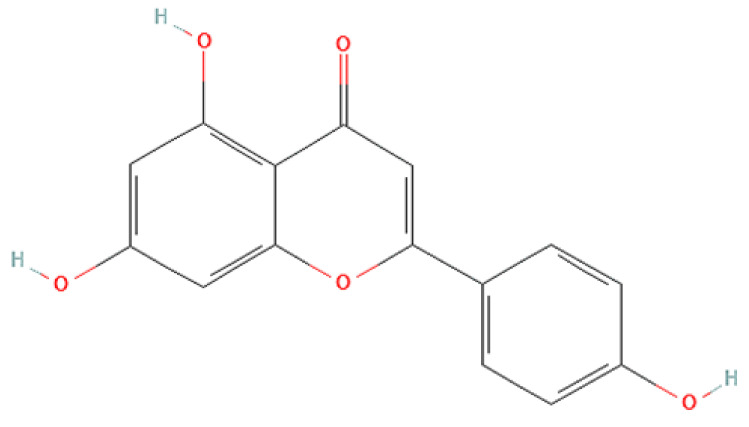	270.053	271.06 (+) 269.04 (−)	35.97	Anti-candidal [43,44,45,46] Anti-hyphal forming activity [46] Anti-biofilm [46] Cytoprotective (retinal cells) [47,48,49]
Asacoumarin A	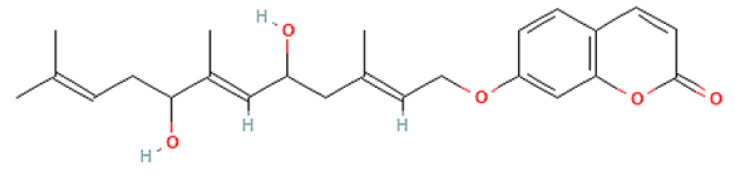	398.209	399.20 (+) 397.20 (−)	37.98	n.a.
Campneoside	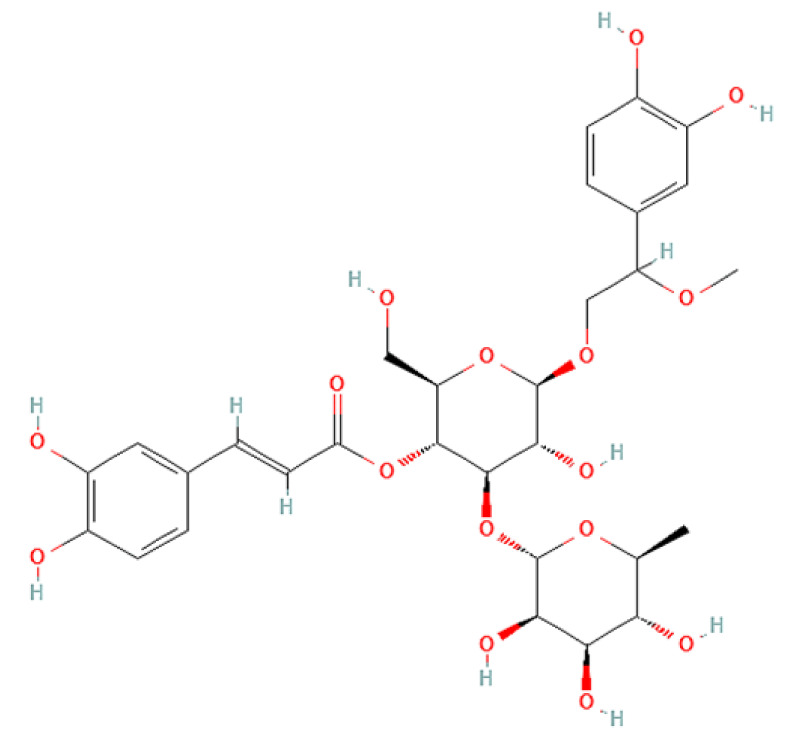	654.216	n.r.	43.27	n.a.
Acutissimin A	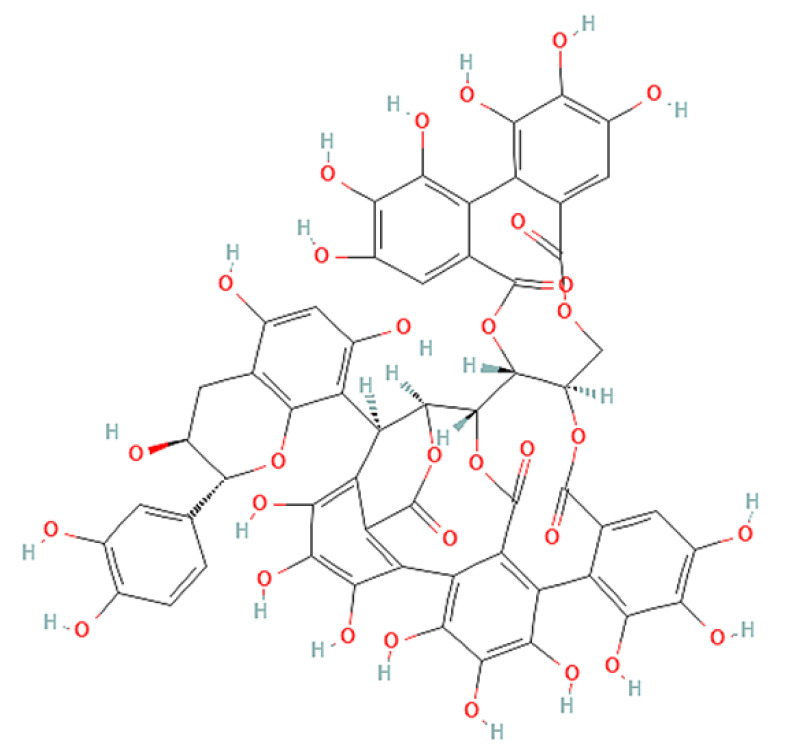	1206.822	1207.14 (+) 1205.10 (−)	n.r.	n.a.
Castacrenin F	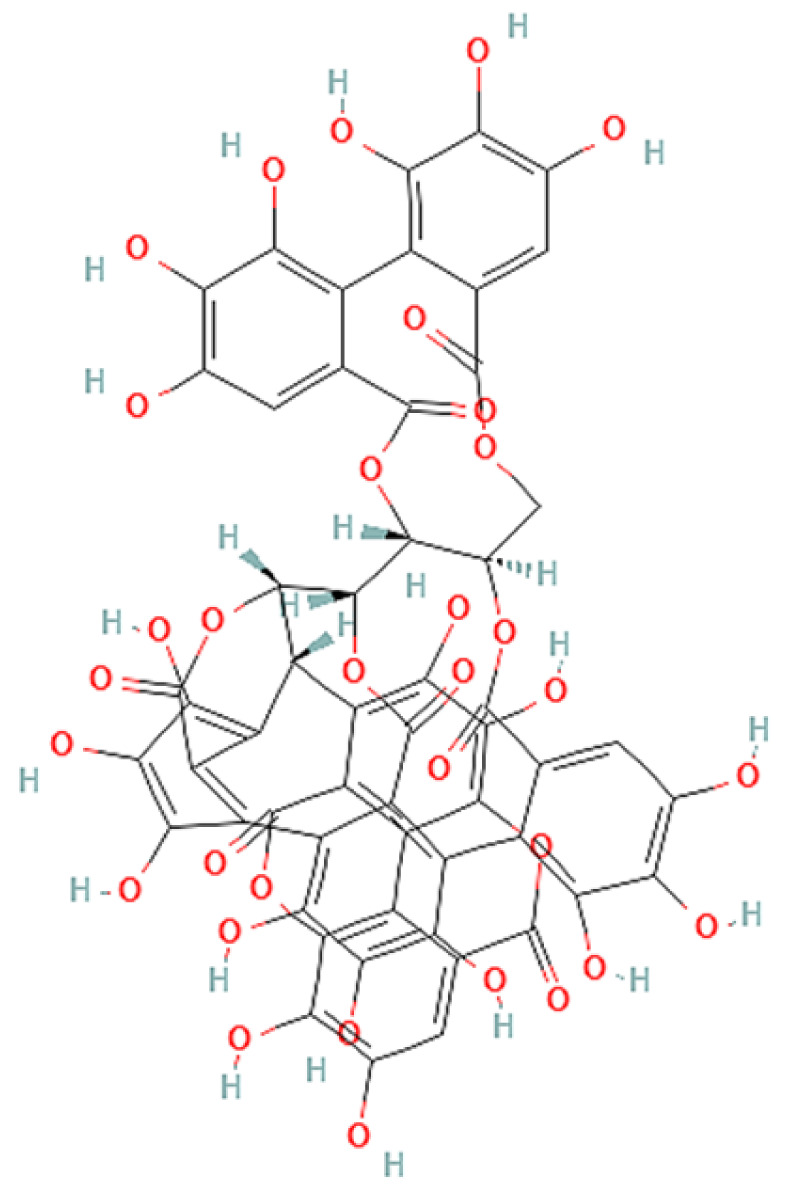	1218.145	1219.07 (+) 1217.10 (−)	n.r.	n.a.
Castacrenin C	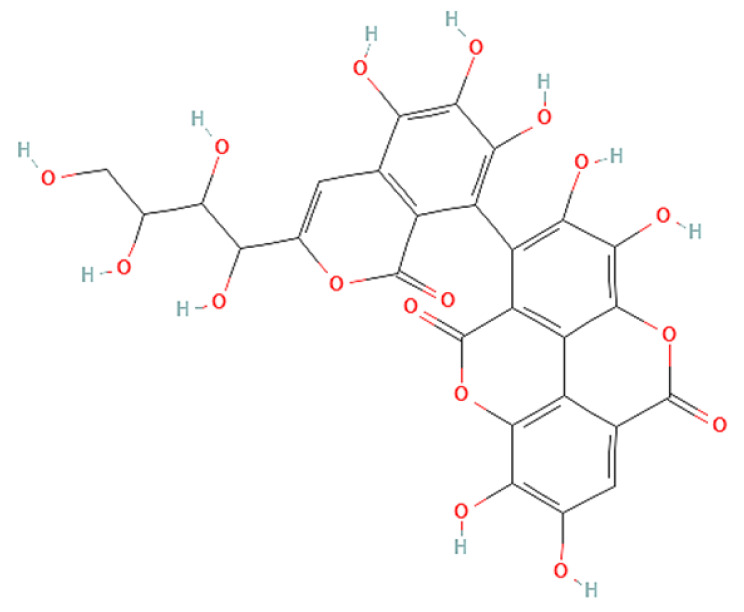	614.054	615.06 (+) 613.05 (−)	36.96	n.a.
Castacrenin E	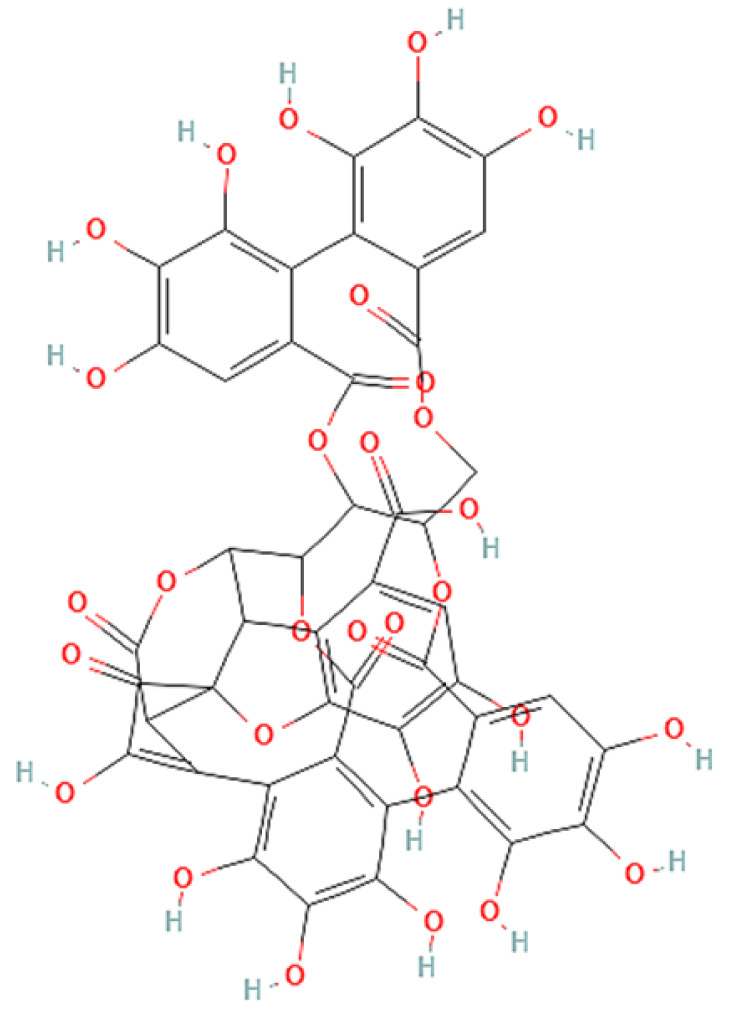	1056.072	1057.07 (+) 1055.06 (−)	32.20	n.a.
Chesnatin	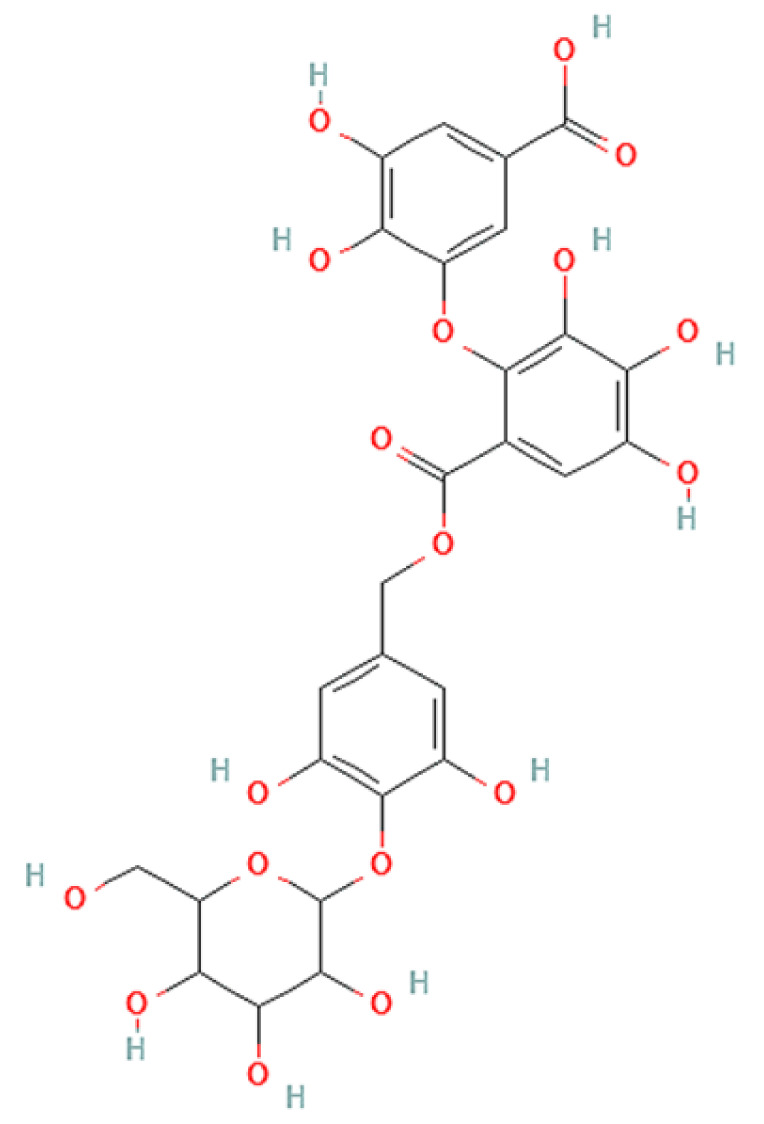	638.112	639.11 (+) 637.11 (−)	28.46	n.a.
Chestanin	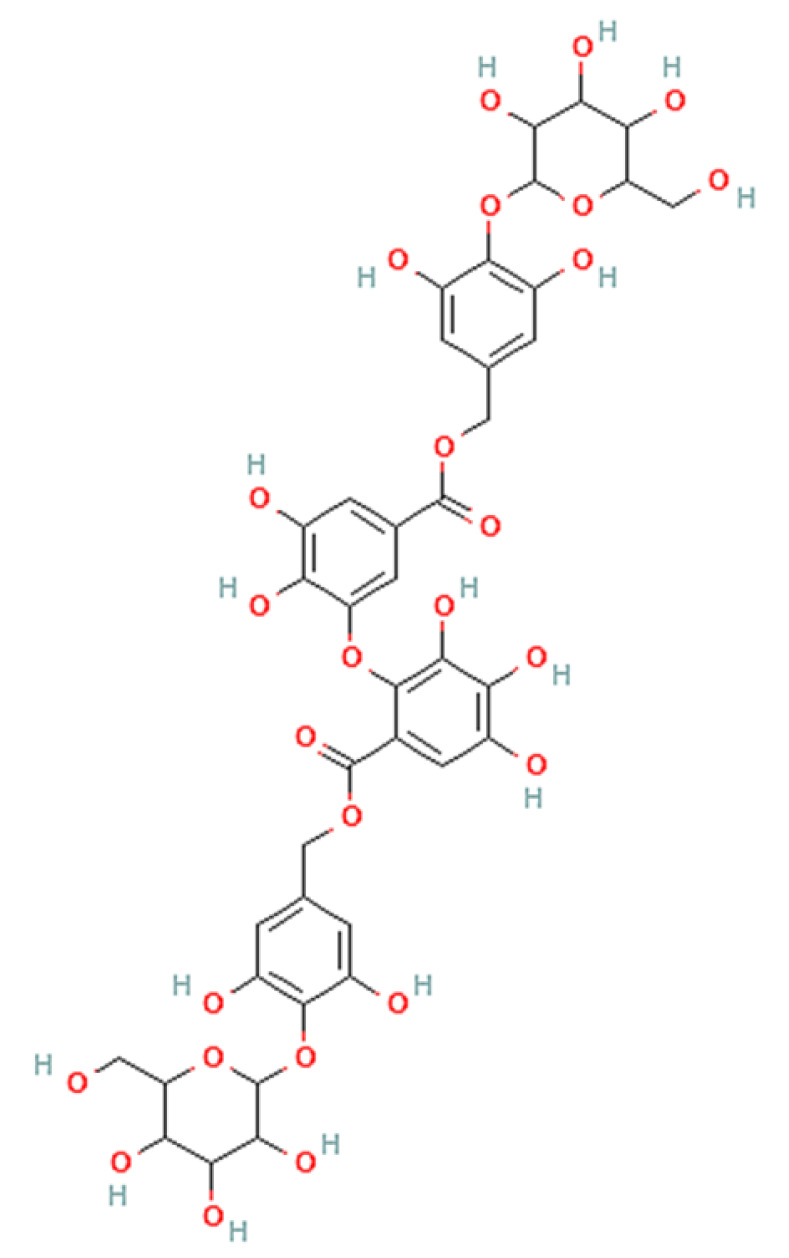	938.196	939.20 (+) 937.18 (−)	43.62	n.a.
Crenatoside	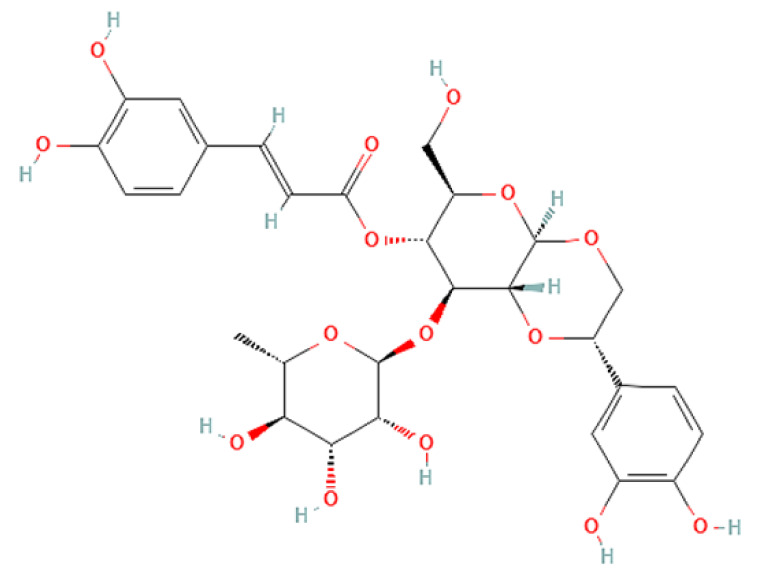	622.190	n.r.	54.02	n.a.
Dibutyl disulfide	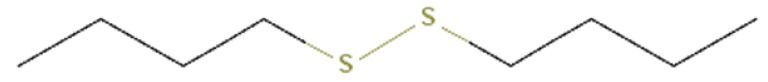	178.085	179.09 (+) 177.07 (−)	39.53	n.a.
Kurigalin	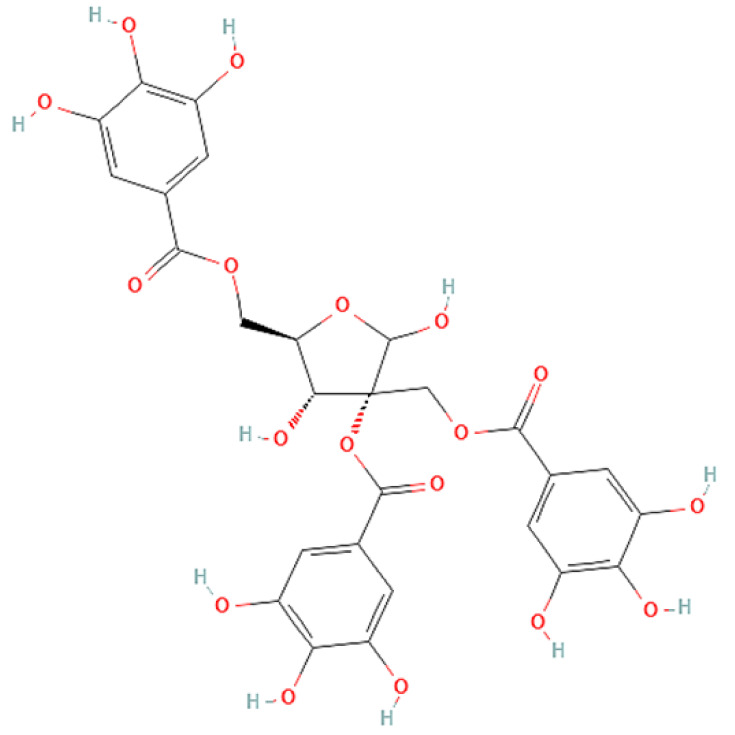	636.096	637.10 (+) 635.10 (−)	44.66	n.a.
Leucosceptoside A	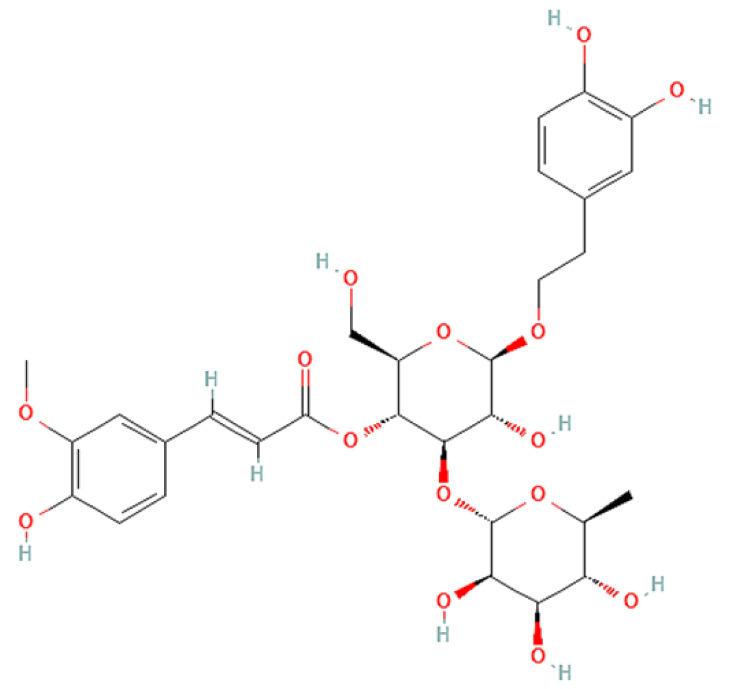	638.221	n.r.	38.38	n.a.
Luteolin	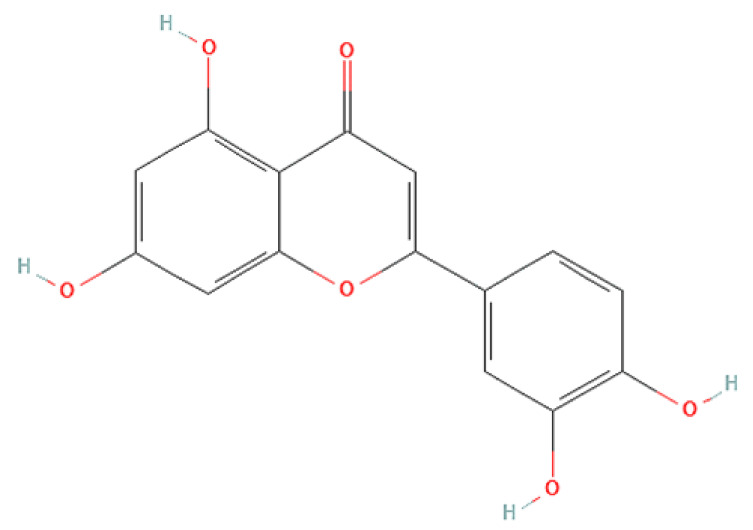	286.048	287.05 (+) 285.03 (−)	21.85	Anti-candidal [46] Anti-hyphal forming activity [46] Anti-biofilm [46] Cytoprotective (retinal cells) [50,51,52,53,54]
Nevskin	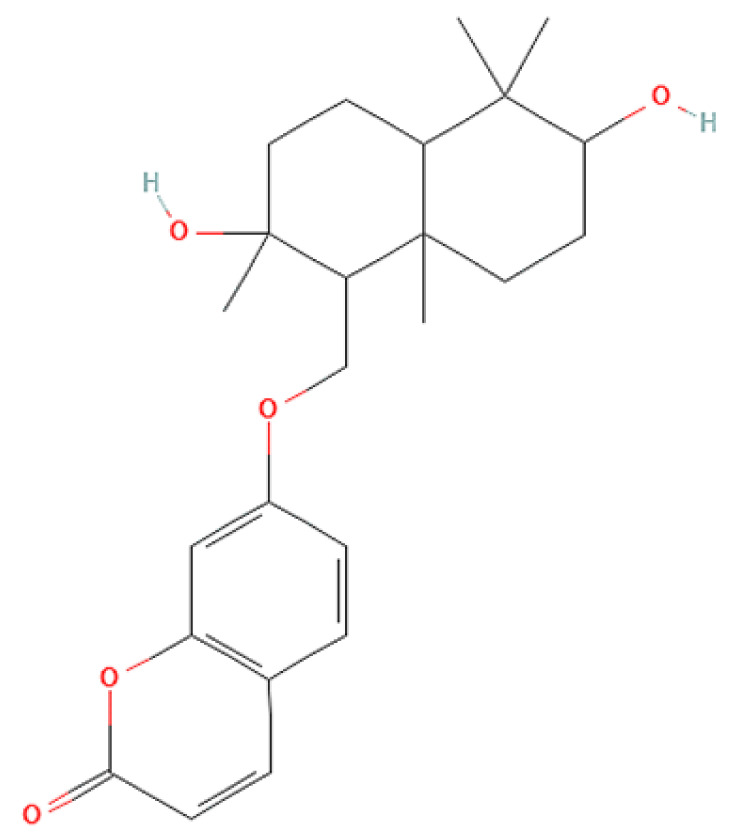	400.225	401.23 (+) 399.20 (−)	14.06	n.a.
Salidroside	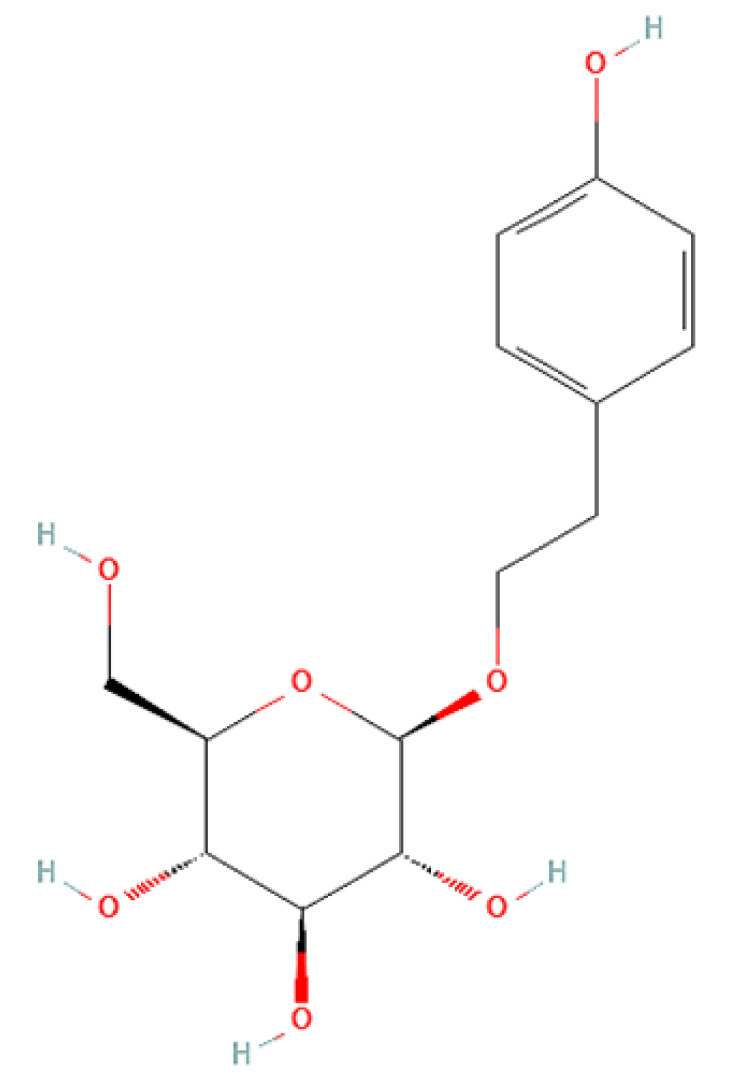	300.121	301.12 (+) 299.10 (−)	35.10	Cytoprotective (retinal cells) [55,56,57,58]

Notes: * RT: retention time; +: positive polarity, −: negative polarity; n.r.: not reported (the retention time of some compounds was not reported, due to their low concentrations in the extract); n.a.: no anti-candidal, anti-biofilm, anti-hyphal, and cytoprotective activities.

## Data Availability

The data presented in this study are available on request from the corresponding author.

## Supplementary Materials

The following are available online https://www.mdpi.com/article/10.3390/antibiotics10111373/s1, Figure S1: Tandem mass spectrum of *O. crenata* leaf extract in positive ion mode (m/z 100–1500 Da), Figure S2: Tandem mass spectrum of *O. crenata* leaf extract in positive ion mode (m/z 100–300 Da), Figure S3: Tandem mass spectrum of *O. crenata* leaf extract in positive ion mode (m/z 300–700 Da), Figure S4: Tandem mass spectrum of *O. crenata* leaf extract in positive ion mode (m/z 700–1100 Da), Figure S5: Tandem mass spectrum of *O. crenata* leaf extract in positive ion mode (m/z 1100–1500 Da), Figure S6: Tandem mass spectrum of *O. crenata* leaf extract in negative ion mode (m/z 100–1500 Da), Figure S7: Tandem mass spectrum of *O. crenata* leaf extract in negative ion mode (m/z 100–300 Da), Figure S8: Tandem mass spectrum of *O. crenata* leaf extract in negative ion mode (m/z 300–700 Da), Figure S9: Tandem mass spectrum of *O. crenata* leaf extract in negative ion mode (m/z 700–1100 Da), Figure S10: Tandem mass spectrum of *O. crenata* leaf extract in negative ion mode (m/z 1100–1500 Da), Figure S11: Chromatogram of *O. crenata* leaf extract by UPLC-Ms/Ms in positive ion mode, Figure S12: Chromatogram of *O. crenata* leaf extract by UPLC-Ms/Ms in negative ion mode.

## Sample Availability

Samples used in this study are not available.
