# Supplementary material for: Anti-Candidal Activity of the Parasitic Plant Orobanche crenata Forssk
Source: Antibiotics (Basel). 2021 Nov 9;10(11):1373. doi: 10.3390/antibiotics10111373 (PMC8615231; doi:10.3390/antibiotics10111373)
Supplement: Supplementary file 1 [file antibiotics-10-01373-s001.zip › antibiotics-1461296-supplementary.pdf]

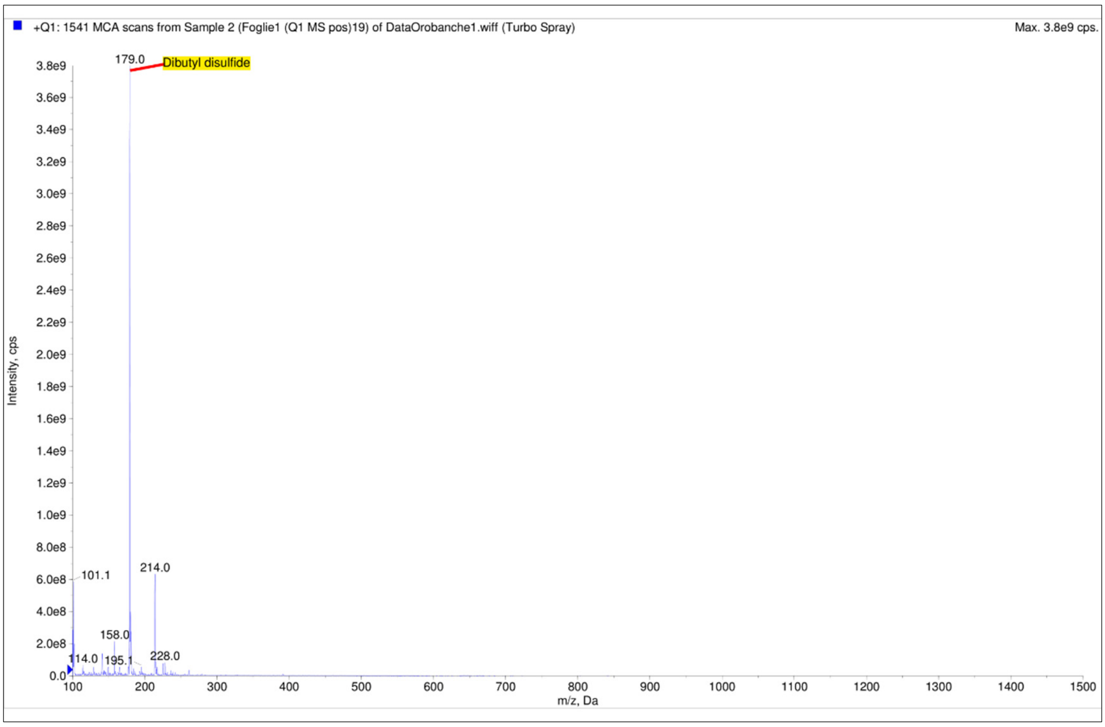

Figure S1. Tandem mass spectrum of *O. crenata* leaf extract in positive ion mode (m/z 100-1500 Da).

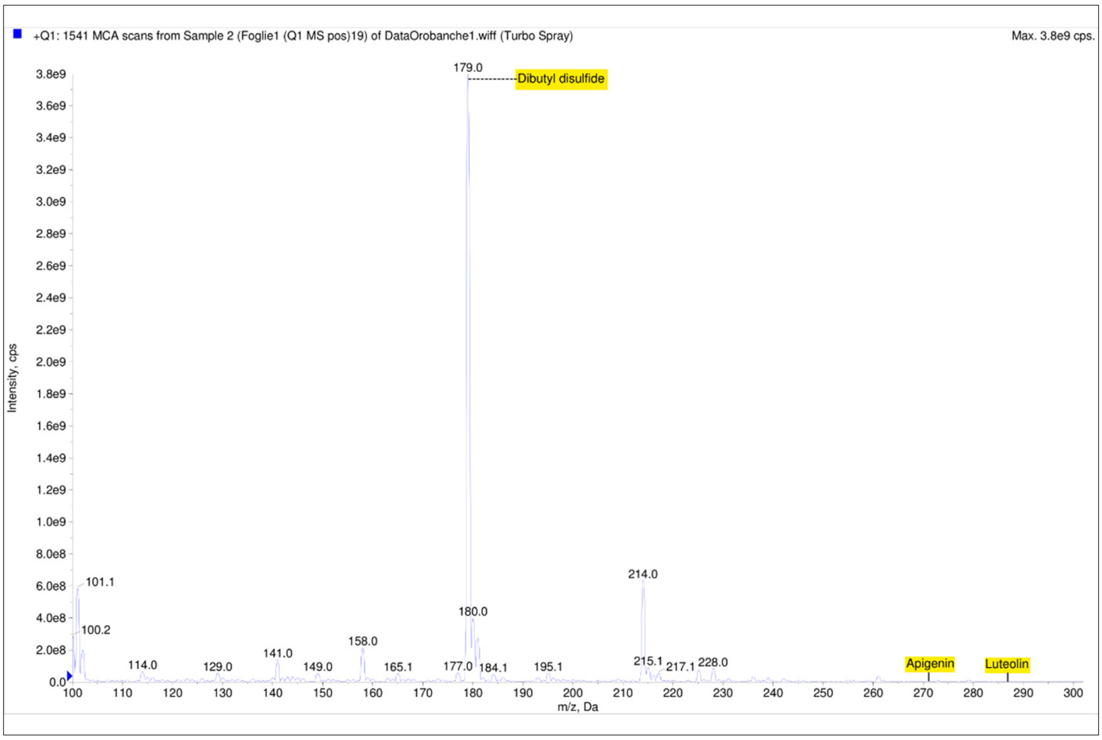

Figure S2. Tandem mass spectrum of *O. crenata* leaf extract in positive ion mode (m/z 100-300 Da).

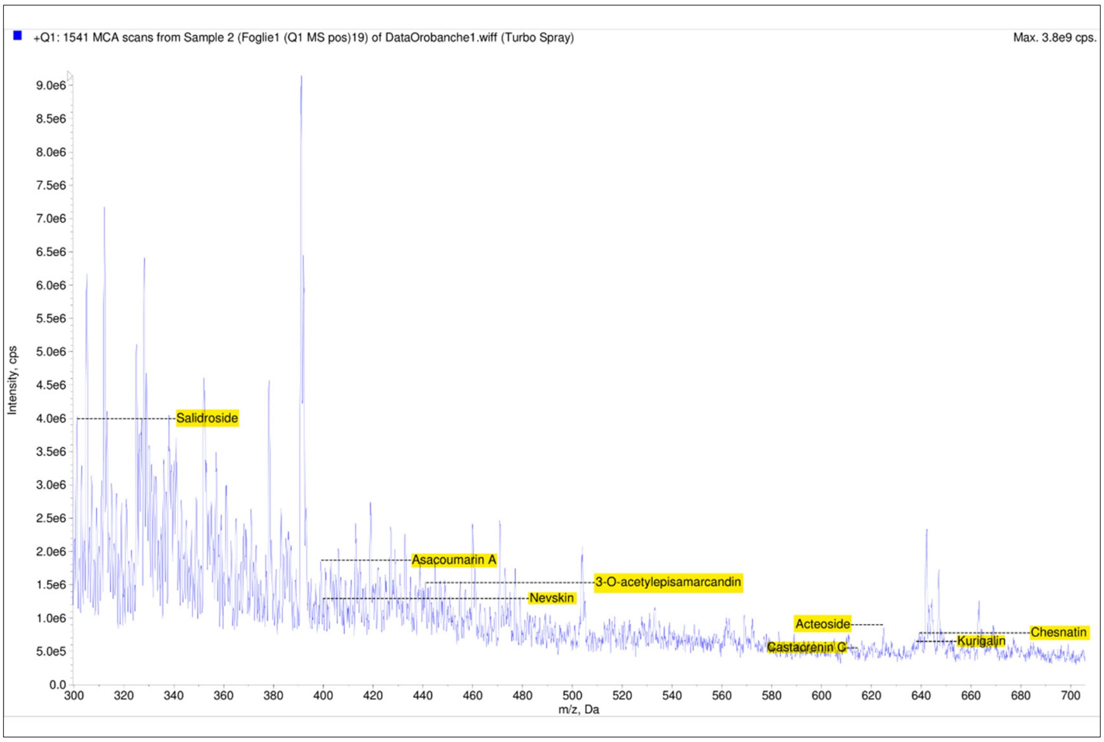

Figure S3. Tandem mass spectrum of *O. crenata* leaf extract in positive ion mode (m/z 300-700 Da).

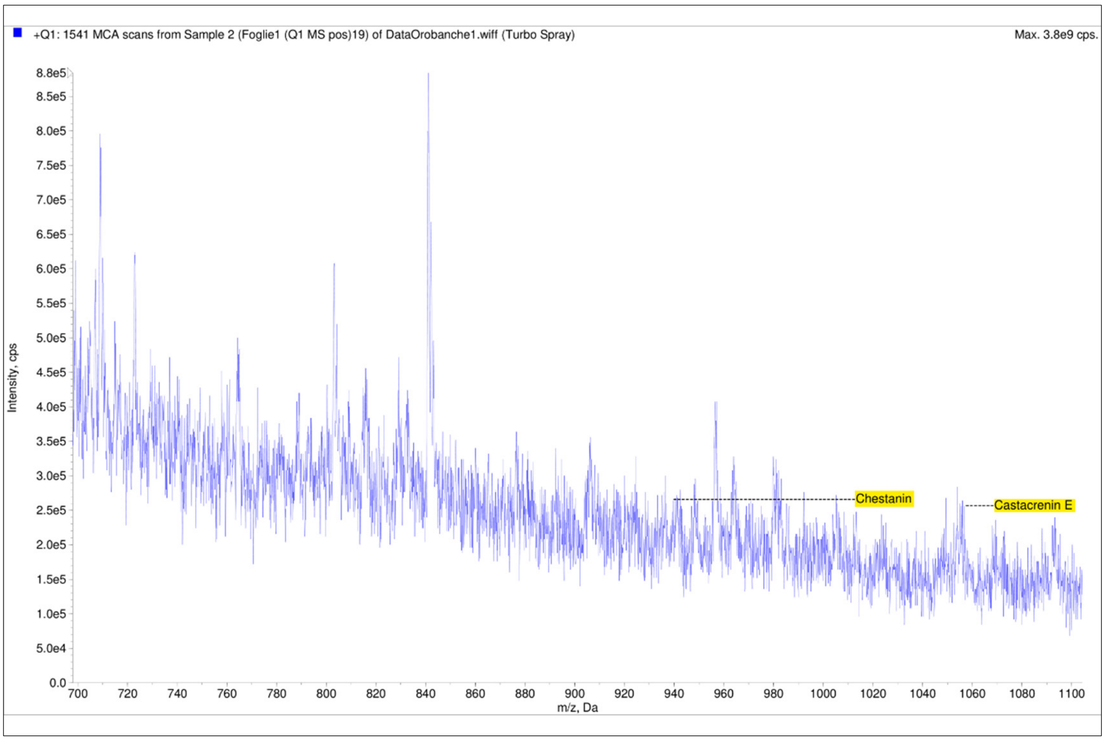

Figure S4. Tandem mass spectrum of *O. crenata* leaf extract in positive ion mode (m/z 700-1100 Da).

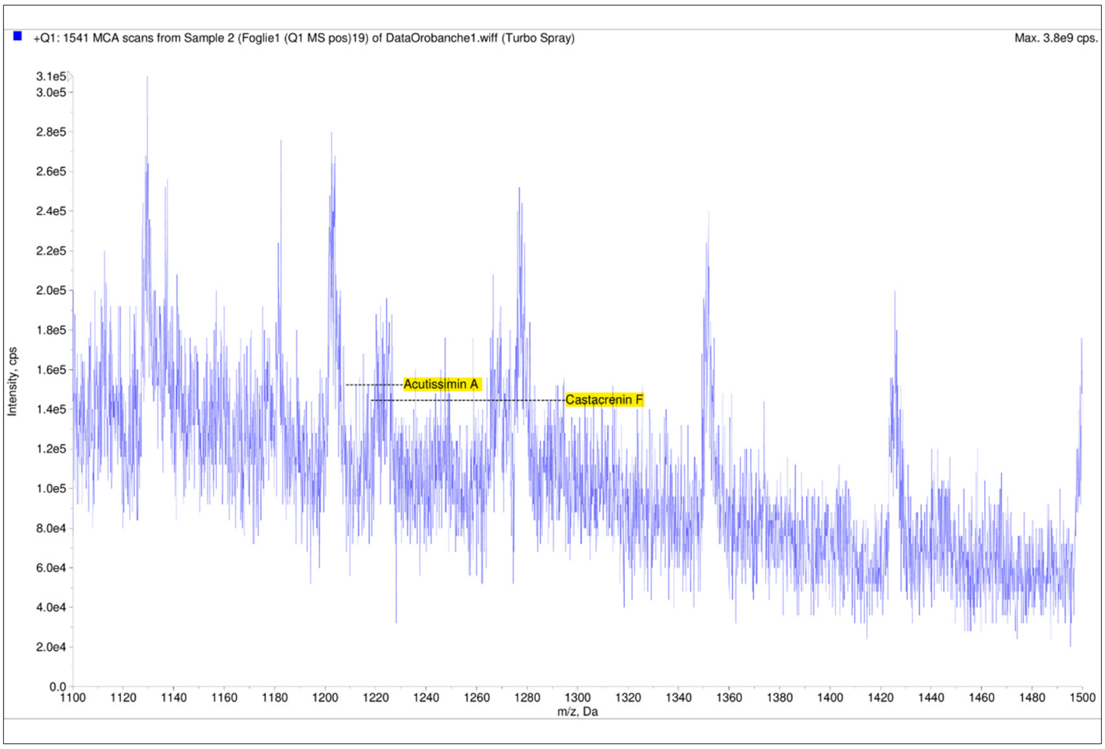

Figure S5. Tandem mass spectrum of *O. crenata* leaf extract in positive ion mode (m/z 1100-1500 Da).

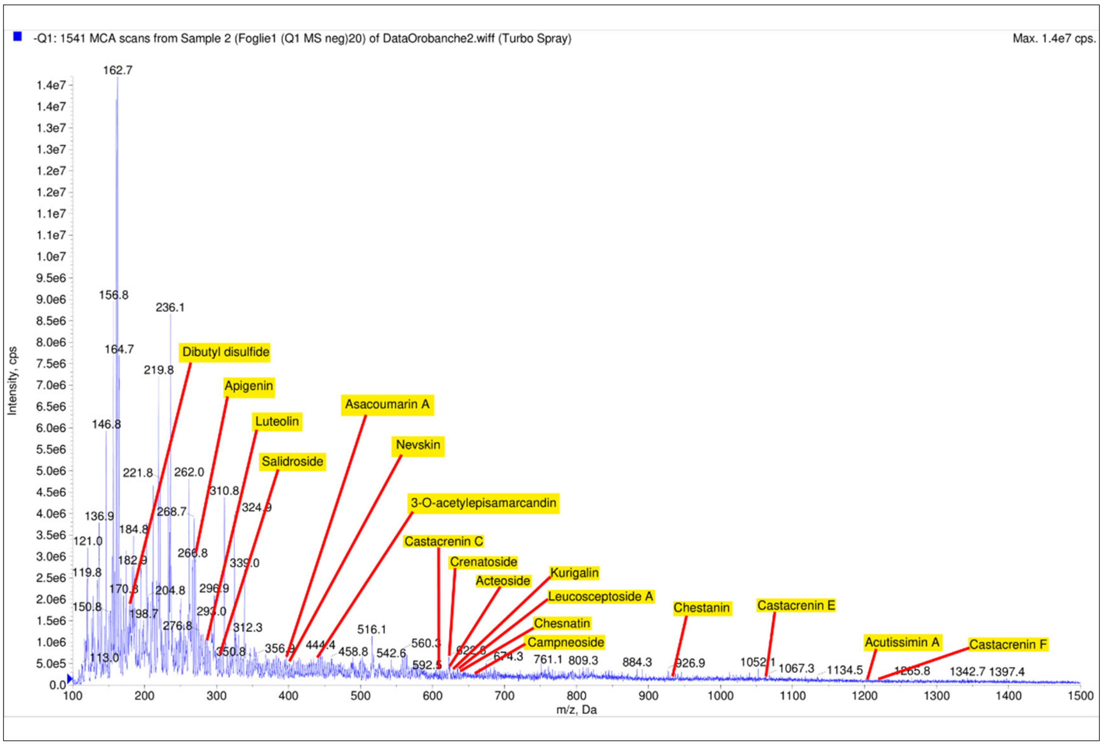

Figure S6. Tandem mass spectrum of *O. crenata* leaf extract in negative ion mode (m/z 100-1500 Da).

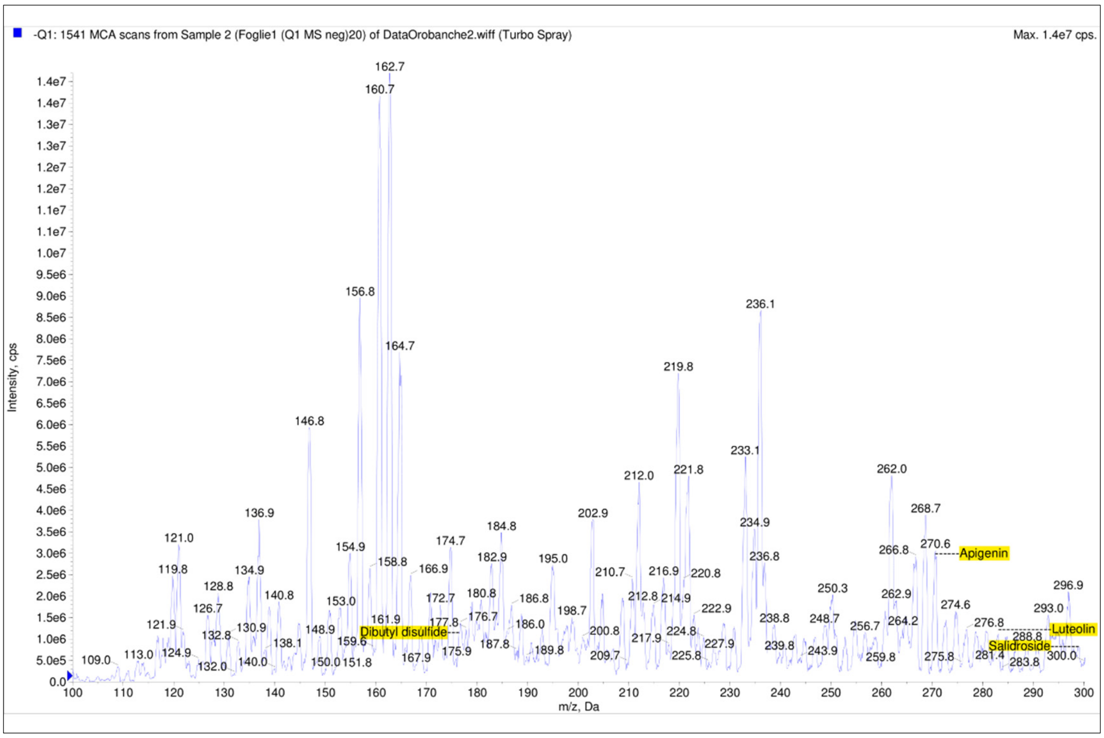

Figure S7. Tandem mass spectrum of *O. crenata* leaf extract in negative ion mode (m/z 100-300 Da).

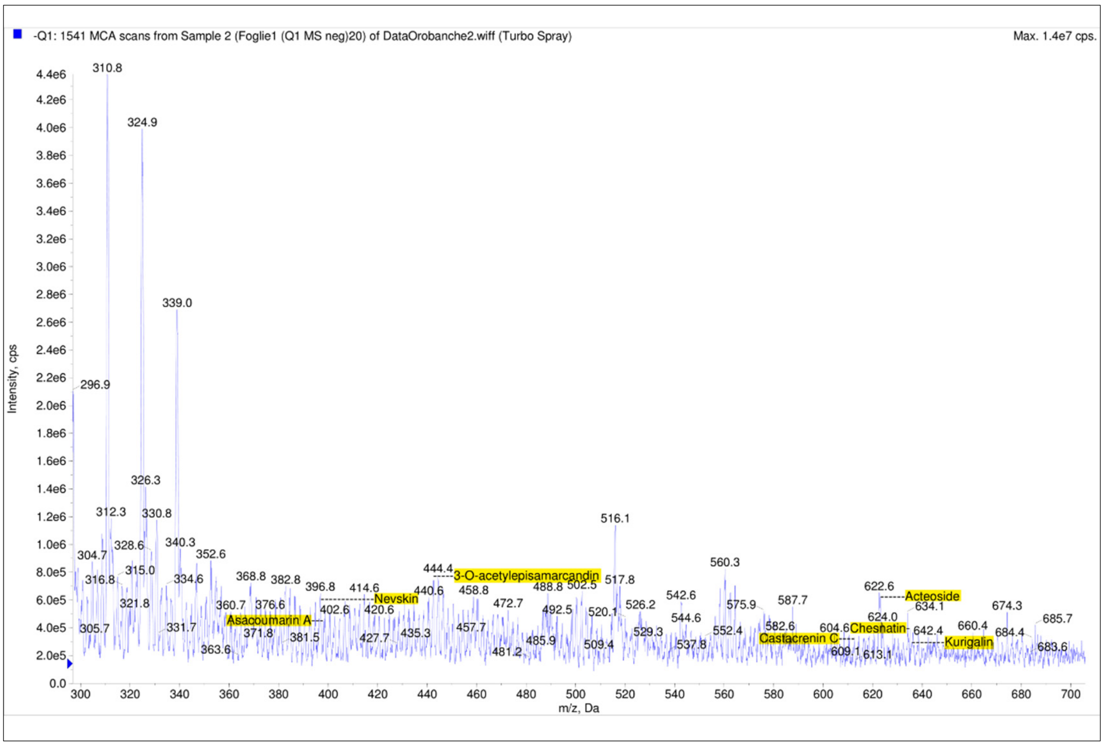

Figure S8. Tandem mass spectrum of *O. crenata* leaf extract in negative ion mode (m/z 300-700 Da).

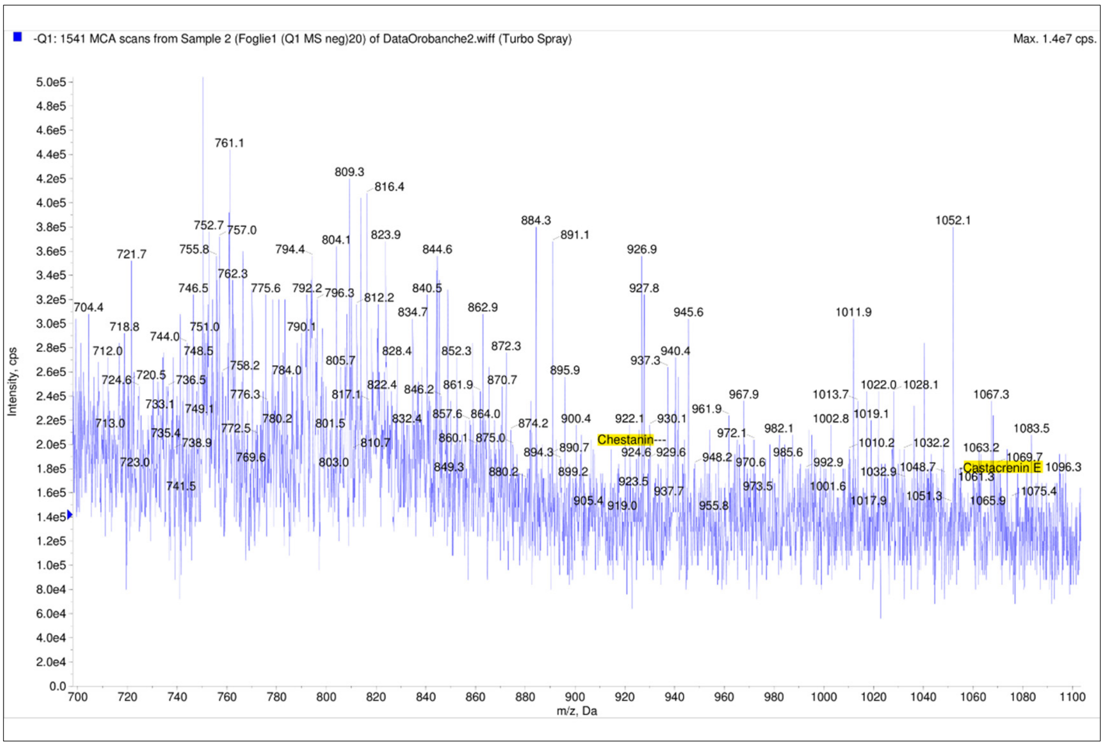

Figure S9. Tandem mass spectrum of *O. crenata* leaf extract in negative ion mode (m/z 700-1100 Da).

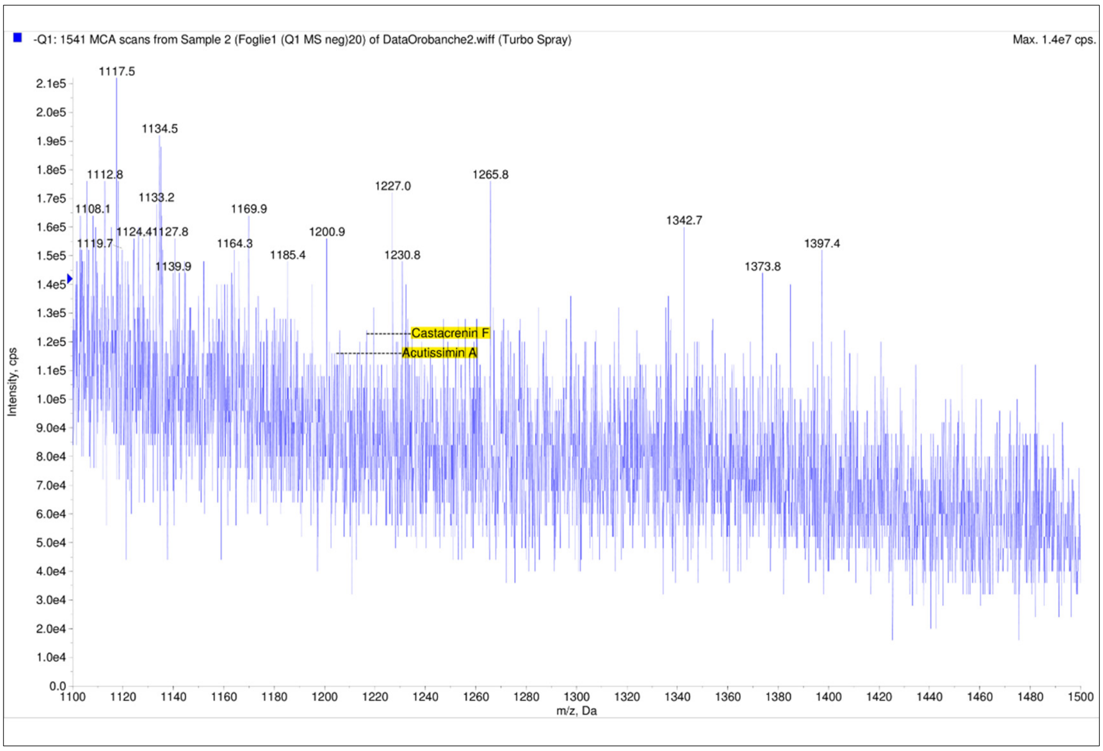

Figure S10. Tandem mass spectrum of *O. crenata* leaf extract in negative ion mode (m/z 1100-1500 Da).

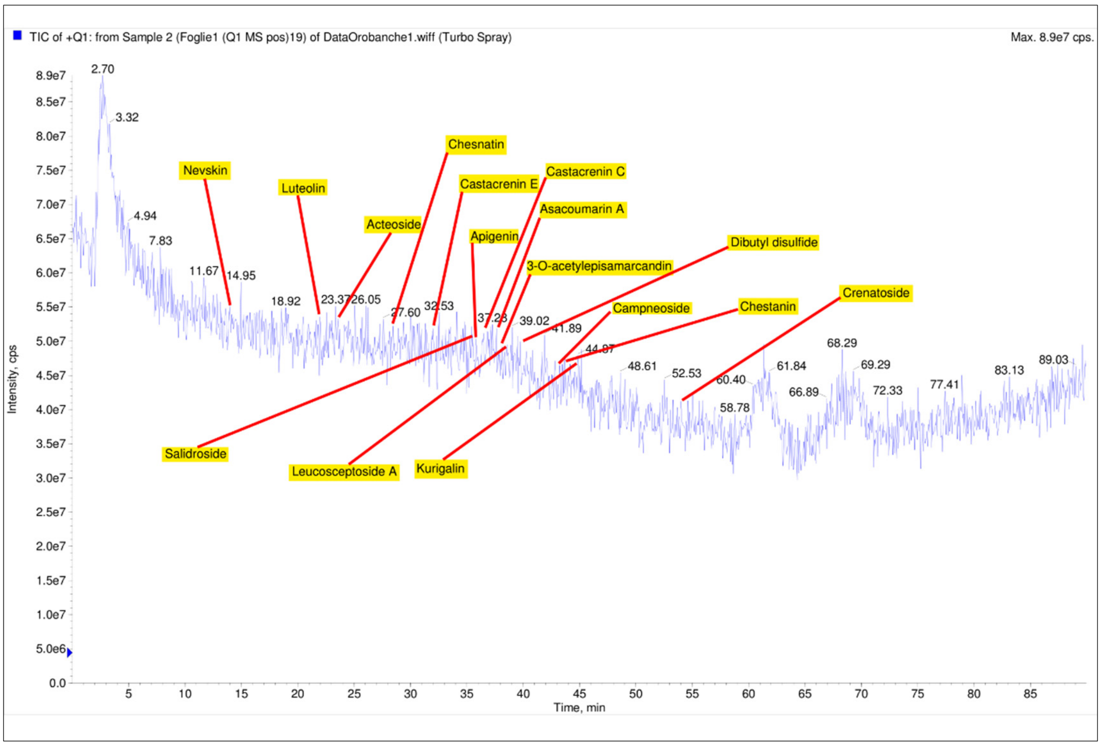

Figure S11. Chromatogram of *O. crenata* leaf extract by UPLC-Ms/Ms in positive ion mode.

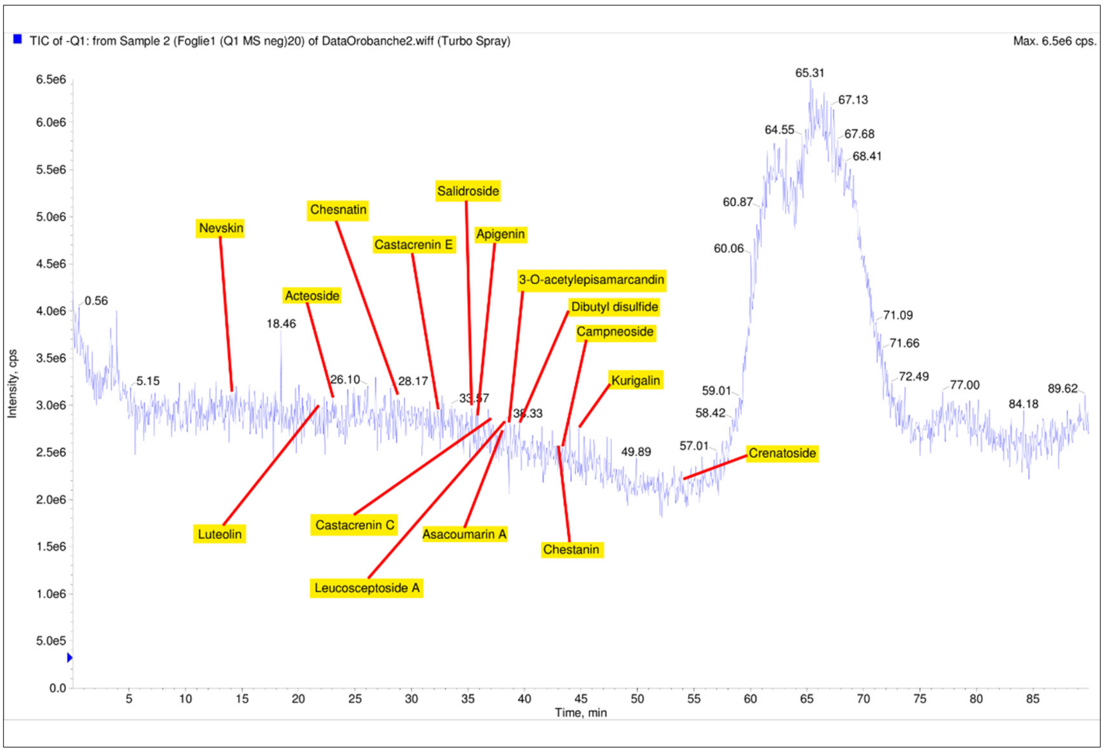

Figure S12. Chromatogram of *O. crenata* leaf extract by UPLC-Ms/Ms in negative ion mode.
